# Risky Outdoor Play and Adventure Education in Nature for Child and Adolescent Wellbeing: A Scoping Review

**DOI:** 10.3390/bs16010005

**Published:** 2025-12-19

**Authors:** Tonia Gray, Michael J. A. Down, Jeff Mann, Jaydene Barnes, Marion Sturges, David Eager, Fiona Pigott, Alexandra Harper, Susan Hespos, Robyn Monro Miller, Arianne Reis

**Affiliations:** 1 Centre for Educational Research, School of Education, Western Sydney University, Sydney 2751, Australia; t.gray@westernsydney.edu.au; 2 World Leisure Centre for Excellence, Western Sydney University, Sydney 2751, Australia; j.barnes@westernsydney.edu.au (J.B.); m.sturges@westernsydney.edu.au (M.S.); f.pigott@westernsydney.edu.au (F.P.); a.reis@westernsydney.edu.au (A.R.); 3 School of Education, Western Sydney University, Sydney 2751, Australia; j3ffmann1@gmail.com; 4 School of Education, The University of Notre Dame Australia, Fremantle 6160, Australia; 5 Faculty of Engineering and Information Technology, University of Technology Sydney, Sydney 2007, Australia; david.eager@uts.edu.au; 6 MARCS Institute of Brain, Behaviour and Development, Western Sydney University, Sydney 2750, Australia; s.hespos@westernsydney.edu.au; 7 Play Australia, Melbourne 3006, Australia; 8 International Play Association, London SN6 8TY, UK; 9 School of Health Sciences, Western Sydney University, Penrith 2751, Australia

**Keywords:** risk, child and adolescent development, protective factors, self efficacy, learning, social determinants of health

## Abstract

According to the Australasian Society for Developmental Paediatrics, experiential learning and outdoor play contain elements of risk, bravery, uncertainty, exploration, personal challenge, and adventure. These attributes are fundamental to a child’s growth, development, and wellbeing, and yet, in contemporary society, outdoor experiences have significantly declined. This scoping review explores the benefits and affordances of nature-based risky play and adventure education across early childhood and adolescence, asking what developmental opportunities emerge when children and adolescents engage in meaningful outdoor challenges. Adopting a benefit–risk approach where safety is “as safe as necessary” rather than “as safe as possible,” the review identifies common elements across developmental stages. A scoping review following PRISMA-ScR guidelines synthesised empirical studies (2015–2025). Our review included 40 empirical studies from a total of 5218 references, using diverse methodologies conducted predominantly in Western nations. All 40 studies reported positive associations across multiple developmental domains. Eight key themes developed: resilience and confidence, wellbeing, physical skills, autonomy and agency, nature connectedness, quality play provision, and educator influence. Authentic child agency and autonomy functioned as critical mechanisms through which benefits are realised across early childhood and school-aged populations. Key benefits included enhanced mental health, social competence, and anxiety prevention. Implementation barriers persist, including parental anxiety, institutional liability concerns, and cultural risk aversion. Evidence overwhelmingly supports nature-based risky play and outdoor adventure education as beneficial for child and adolescent development. Translation into practice remains limited by stakeholder attitudes and systemic barriers. Future research should prioritise longitudinal studies, cross-cultural investigation, and equity-focused approaches addressing disparities in access to positive risk-taking.

## 1. Introduction

Child and adolescent risk-taking and outdoor adventure have undergone dramatic changes over recent decades (T. Gray et al., 2025a; Haidt, 2024). Traditional forms of unstructured and challenging activities, such as risky play, have been replaced by highly structured, adult supervised, and risk-averse activities (Charan et al., 2024; Eager et al., 2025; Loebach et al., 2021; Sturges et al., 2023). This shift has occurred alongside escalating concerns about overreliance on digital devices and screen time, physical inactivity, rising mental health challenges, and other associated developmental outcomes for children and youth (Australasian Society for Developmental Paediatrics, ASDP, 2025; Beaulieu & Beno, 2024; Dodd et al., 2023; T. Gray et al., 2025a; Liu et al., 2025; Scott et al., 2022). In the same vein, P. Gray et al. (2023) provide compelling evidence that the decline in self-directed and independent activity has contributed to the deterioration in a child’s mental wellbeing. Haidt (2024) purported that excessive screen exposure is linked to a marked decline in resilience, self-regulation, physical activity, creativity, and face-to-face social interaction. Given these troubling findings, he coined the term “Gen Overwhelmed” or “Gen Anxious” for the youth of our times.

With the advent of “helicopter parenting” in recent decades, T. Gray et al. (2025a) argued that “modern society is at a dangerous crossroads, preoccupied with hermetically sealing children from harm or risk exposure yet, paradoxically, creating a deficit in mental toughness and resilience” (p. 37). De Lannoy (2024) documented the transformation in outdoor play opportunities, while Loebach et al. (2021) identified socioenvironmental barriers to community-based outdoor play. Most alarmingly in this digital era, Charan et al. (2024) described the need to rescue children’s outdoor playtime for their holistic development whether it be physical health, cognitive stimulation, or socioemotional wellbeing.

Within the milieu of declining opportunities, risky play emerges not merely as a lost practice but as a critical developmental necessity. The publication of the international standard on benefit–risk allowed for societal acceptance of risk in outdoor recreation activities and education (Eager, 2024). This need has recently been highlighted by position statements on risky play from two international paediatric societies: the ASDP (2025) and the Canadian Paediatric Society (Beaulieu & Beno, 2024). Early experiences shape children’s brain development and set strong foundations for adolescence and lifelong health and wellbeing. Risk-taking develops children and adolescents’ executive function skills, risk intelligence, and risk literacy, which includes the ability to adjust their behaviour and mindset to achieve goals, as well as emotional regulation and problem-solving skills (Eager et al., 2025; T. Gray et al., 2025a).

Evidence-based research suggests that exposure to risk-taking in activities such as nature-based play and adventure outdoor education serves as an important catalyst for our developmental functions across multiple domains (Down et al., 2022; Eager et al., 2025; Harper et al., 2025). In turn, this helps children and youth overcome fears and develop resilience (T. Gray et al., 2025a; Sandseter et al., 2023b). Contemporary research repeatedly demonstrates a positive association between healthy risk-taking and physical and mental wellbeing, social competence, and cognitive development (T. Gray et al., 2025c; Liu et al., 2025; McCallum et al., 2024; Sando et al., 2021; Sturm et al., 2025). Based on this evidence, Meijer et al. (2024) advocated for planning approaches that move from child-safe playgrounds towards adventurous urban areas. However, despite growing recognition of these benefits, children and adolescents’ opportunities for risky play and adventurous activities have declined significantly due to increased parental anxiety, institutional liability concerns, cultural shifts towards risk aversion, and environmental changes (T. Gray, 2018; Jones et al., 2025; Oliver et al., 2023; Sturges et al., 2023).

This scoping review explores the benefits of risk-taking in nature play and adventurous outdoor activities for children and adolescents. It differs from previously published reviews in that its primary focus is on activities engaged in natural environments. The primary aim of this scoping review is to identify the health outcomes of nature-based risky play and adventurous activities for children and youth up to 18 years of age.

## 2. Definitions and Conceptualisations of Risky Play and Outdoor Adventure Education

The conceptualisation of risky play as a distinct research area began when Sandseter (2007) established a foundational taxonomy identifying six categories: (1) play with great heights, (2) play with high speed, (3) play with harmful tools, (4) play near dangerous elements, (5) rough-and-tumble play, and (6) play where children can “disappear” or get lost. This taxonomic framework provided the influential definition of risky play as thrilling and exciting forms of play that involve a risk of physical injury. Risky play primarily takes place outdoors, often as challenging and adventurous physical activities, children attempting something they have never done before, skirting the borderline of the feeling of being out of control (often because of height or speed) and overcoming fear (Sandseter & Kennair, 2011).

Subsequent research by Kleppe et al. (2017) expanded this framework to eight categories by adding impact and vicarious risk while adapting definitions for younger children as “play that involves uncertainty and exploration—bodily, perceptual or environmental—that could lead to negative consequences” (p. 18). Stevens-Smith and Murdock (2020) identified seven foundational play elements—balancing, sliding, brachiating, spinning, climbing, swinging, and sensory development—underscoring play’s role in enhancing cognitive, social, and emotional development when appropriately implemented.

Contemporary frameworks have evolved beyond purely physical risk considerations. Sandseter et al. (2023a) expanded conceptualisation to encompass three biopsychosocial levels: mental health and emotion regulation, social functioning and challenging norms, and physical health and development. Recent definitions emphasise agency and positive outcomes, with Jones et al. (2025) characterising risky play as “respectful, exploratory and challenging with often unpredictable outcomes”; that is, “child-initiated and physically engages all the senses leading to positive learning and development outcomes” (p. 7).

Outdoor adventure education (OAE) encompasses a range of pedagogical activities and experiences that engage directly with natural settings and often include either real or perceived risks (Ewert, 2014). Research indicates that risk-taking is driven by intrinsic motivation that is self-initiated and self-controlled (Kvalnes & Sandseter, 2023). This intrinsic motivation appears to align with self-determination theory, which identifies autonomy, competence, and relatedness as fundamental psychological needs that drive human behaviour and development (Ryan & Deci, 2000, as cited in Johnstone et al., 2022).

Extensive research also reveals that when children and adolescents engage in positive risk-taking, they exercise autonomy by making independent choices about challenge levels (Eager et al., 2025; T. Gray, 2019; Sandseter et al., 2023a; Hattie et al., 1997; Little, 2022). Correspondingly, they develop competence through mastering new skills and overcoming fears, and they build relatedness through shared risk-taking experiences with peers (T. Gray et al., 2025c; Pigott et al., 2025; Sando et al., 2021; Sturges et al., 2023). Positive risk-taking fosters skills such as problem-solving, benefit–risk assessment, and self-efficacy, leading to increased self-confidence, mental wellbeing, and decision-making skills. Studies from nature-based educational settings suggest that fulfilment of these basic psychological needs serves as a mediating factor for motivation and wellbeing outcomes, with natural environments supporting children’s sense of autonomy and competence as learners (Harper et al., 2025; Johnstone et al., 2022).

Armstrong et al. (2025) clarified that positive risk-taking “denotes a situation whereby a child can recognise and evaluate a challenge and decide on a course of action” (p. 2), positioning children as active agents rather than passive recipients of environmental dangers. This decision-making process appears to reflect the core principles of self-determination theory, where children’s and adolescents’ autonomous choices in risky situations may support their fundamental needs for competence and self-efficacy (Ryan & Deci, 2000, as cited in Johnstone et al., 2022). Research suggests that, through risky play and adventurous activities, participants actively construct their own learning experiences, potentially developing confidence in their ability to assess and handle challenging situations (Mann et al., 2022; Pigott et al., 2025). Against this backdrop, wellbeing is enhanced when educators “provide well-planned and challenging outdoor environments that encourage risk-taking and risky play experiences” (Speldewinde, 2025, p. 47).

Theoretical foundations for risky play’s mental health benefits originated with Sandseter and Kennair’s (2011) antiphobic theory. Consequently, the positioning of risky play has now evolved to a point where it is “a mechanism to remove remaining nonfunctional anxiety as the child matures and becomes competent to master situations that previously were beyond their capacity to cope with” (Sandseter et al., 2023a, p. 130). This framework suggests that children’s engagement with fearful situations through play serves as natural exposure therapy, gradually building confidence and reducing anxiety responses to environmental challenges.

Dodd and Lester (2021) extended this theoretical understanding by proposing that adventurous play functions as a specific mechanism for reducing childhood anxiety risk. Their conceptual model suggests that activities such as tree climbing, fast downhill cycling, and rock jumping create controlled opportunities for children to experience manageable fear within a positive, self-directed context. According to their framework, the combination of thrilling emotions with playful engagement allows children to develop critical skills for managing physiological arousal, tolerating uncertainty, and building coping competencies. The model proposes that repeated exposure to these manageable challenges progressively strengthens children’s confidence in their ability to handle anxiety-provoking situations while reducing tendencies towards catastrophic thinking about bodily sensations.

Sandseter et al. (2023a) substantially broadened this theoretical foundation, moving beyond anxiety-focused models to establish a comprehensive framework spanning mental health regulation, social norm navigation, and physical development. They argued that risky play serves “a broader emotion regulation function, of which the anti-phobic function is a subcategory” (p. 130). Their evolutionary framework positions children’s attraction to challenging play as an adaptive mechanism driven by excitement and competence building across diverse developmental domains. This reconceptualisation reframes perceived risks as evolutionarily advantageous experiences that promote healthy development rather than merely correcting anxiety-related deficits.

Similarly, recent research reframes adolescent risk-taking as potentially adaptive rather than inherently problematic. Positive risk-taking theory provides foundational understanding, with Duell and Steinberg (2021) distinguishing between positive risks that offer developmental benefits and social acceptability versus negative risks such as substance use and delinquency. Their theoretical model emphasises that positive risk-taking serves essential developmental functions, including identity formation, autonomy establishment, skill development, and opportunity exploration.

The neurobiological development framework offers critical support for positive risk-taking theory. Baker et al. (2025) provided comprehensive findings that puberty initiates significant neurobiological changes that amplify adolescents’ responsiveness to their environment, facilitating neural adaptation. This heightened brain adaptability, combined with adolescents’ social curiosity and appetite for risk, propels them to explore diverse environments and forge social bonds. The authors demonstrate how this exploration accelerates experiential learning and social network formation as adolescents prepare for adult independence.

Experiential learning theory, or “learning by doing,” is the cornerstone of education where knowledge is created through an iterative cycle of experience, reflection, and application (Kolb & Kolb, 2018). According to T. Gray et al. (2025a), experiential learning is described as “an endlessly recurring cyclical process” (p. 48), which supports the continuation of learning processes established in childhood through risky play experiences. Furthermore, these authors provide support that risk-taking experiences serve as catalysts for learning, helping children become better “risk technicians” (p. 44). Baker et al. (2025) extended this framework into adolescence, showing how puberty-related changes in brain reward systems contribute to increases in reward sensitivity, exploration, and risk-taking, which play adaptive roles in learning and cognitive flexibility development.

Adventure education theory is supported by empirical research across multiple studies that demonstrate how structured outdoor experiences can systematically develop resilience and life skills. Mann et al. (2022) provided systematic review findings showing consistent benefits across adventure education programs, including improvements in self-concept, coping factors, resilience, and prosocial behaviours. This theoretical framework emphasises the importance of graduated challenge, environmental immersion, and experiential learning as core mechanisms through which OAE promotes both individual skill development and social cohesion among participants.

The literature reveals how these theoretical frameworks converge around the concept that adolescence represents a critical period in which brain adaptability meets environmental exploration. Baker et al. (2025) stressed that this intersection creates optimal conditions for adaptive learning, with the brain’s ability to adapt and change based on experiences forming the foundation for behaviours necessary for adult independence. Down et al. (2025a, 2025b), and Patterson et al. (2023) provided further empirical support, showing how structured programs can harness these natural developmental tendencies while supporting both physical and social risk-taking that enhances overall development.

Collectively, these unite by recognising risky play as serving multiple adaptive functions across development. Early childhood theories emphasise agency, embodied learning, and anxiety regulation, while adolescent frameworks highlight positive risk-taking and structured adventure experiences (Sisk & Gee, 2022). This theoretical progression suggests that risky play functions evolve but remain crucial throughout development, supporting physical, cognitive, emotional, and social growth through age-appropriate challenge and exploration.

While evidence suggests that risky play and OAE provide developmental benefits for children and youth, there is still societal reluctance to adopt these practices in schools and beyond to support their learning and growth. It seems relevant, therefore, to provide a comprehensive map of the existing evidence on nature-based risky play and OAE in childhood and adolescence to support change in practice.

## 3. Materials and Methods

A preliminary search was conducted across different databases, and systematic or scoping reviews were identified in areas related or tangential to the topic, but no precise overlap was identified. The studies found included a systematic review on the relationship between risky outdoor play and health in children (Brussoni et al., 2015), a systematic review on children’s (Jerebine et al., 2022a) and adults’ (Jerebine et al., 2022b) perceptions of safety and risk in active play in schools, and a systematic review on the impacts of unstructured nature play on health in early childhood development (Dankiw et al., 2020). The current scoping review on the benefits of risky play and adventure education differs from previously published reviews in its focus on risky play and adventure education that happens specifically in natural environments; it intends to map the research in this space, including the perceptions of benefits from all stakeholders involved. The review procedure was registered prospectively with the Open Science Framework on 17 August, in line with the recommendation from the *JBI Manual for Evidence Synthesis* (Aromataris et al., 2024), is presented as per the 2020 Preferred Reporting Items for Systematic reviews and Meta-Analyses extension for Scoping Reviews (PRISMA-ScR) guidelines (Page et al., 2020), and followed the PRISMA-ScR 2020 Item Checklist (Supplementary File S1, Tricco et al., 2018).

### 3.1. Review Questions

The primary aim of this scoping review was to identify the outcomes of nature-based risky play and adventurous activities for children and youth up to 18 years of age. To address this aim, the following research questions were established:

1. What are the physical and mental health outcomes of nature-based risky play and adventurous activities for children and adolescents up to 18 years of age?
2. What are the psychosocial outcomes of nature-based risky play or adventurous activities for children and adolescents up to 18 years of age?

### 3.2. Eligibility Criteria

#### 3.2.1. Population

This scoping review includes studies on the outcomes of nature-based risky play and adventure education for children and adolescents from 0 to 18 years of age. While the focus is on children and young people, perspectives from parents and teachers are also included if they relate to the outcomes of interventions with the focus population.

#### 3.2.2. Phenomenon of Interest (Concept)

The phenomenon of interest was interventions in natural environments that involve a level of risk that challenges participants. The interventions may aim to improve school and learning-related performance measures or improve individual cognitive, psychosocial, or physical characteristics. Interventions in which risk was not a key aspect of the experience were not included.

#### 3.2.3. Context

The natural environment was the key context for the studies included in this review. Outdoor spaces within school grounds or similar were excluded given the significant human-made modifications present in these environments. Playgrounds were excluded for the same reason. Urban or rural parks were included if they were not significantly modified with built structures.

#### 3.2.4. Types of Sources

This scoping review considered all types of empirical studies, including those using quantitative, qualitative, or mixed methods. Text and opinion papers were only considered for inclusion if they presented empirical data. Reviews were not included but were checked for further sources.

#### 3.2.5. Search Strategy

The search strategy aimed to locate both published and unpublished studies. A three-step search strategy was utilised in this review. First, an initial limited search of MEDLINE (PubMed) and CINAHL (EBSCO) was conducted to identify articles on the topic. In consultation with a university librarian who is an expert in this field, the words in the titles and abstracts of relevant articles, along with the index terms used to describe the articles, were used to develop a full search strategy for five databases, which were adapted as required: ERIC (ProQuest), APA PsycInfo (EBSCO), Academic Search Complete (EBSCO), Education Research Complete (EBSCO), and ProQuest Central. Search terms and subject or field codes for each of the databases are listed in Supplementary File S2. Searches were limited to articles published between 2015 and 2025 to ensure currency of sources. Only studies published in English were included due to limited capacity within the team to proficiently assess texts in other languages.

The following databases were searched (with search strings included in parentheses):

- APA PsycInfo, Academic Search Complete, and Education Research Complete (EBSCO): (child* OR infan* OR toddler* OR preschool* OR adol* OR teen* OR youth*) AND (“risky play” OR “adventur*” OR “risk* play”) AND (“physical development” OR “cognitive development” OR “emotional development” OR wellbeing OR “well-being”)
- ERIC and ProQuest Central: Identical terms with appropriate filters
- Citation chasing: Google Scholar and reference list hand-searching

#### 3.2.6. Selection and Data Extraction

**Figure 1:**
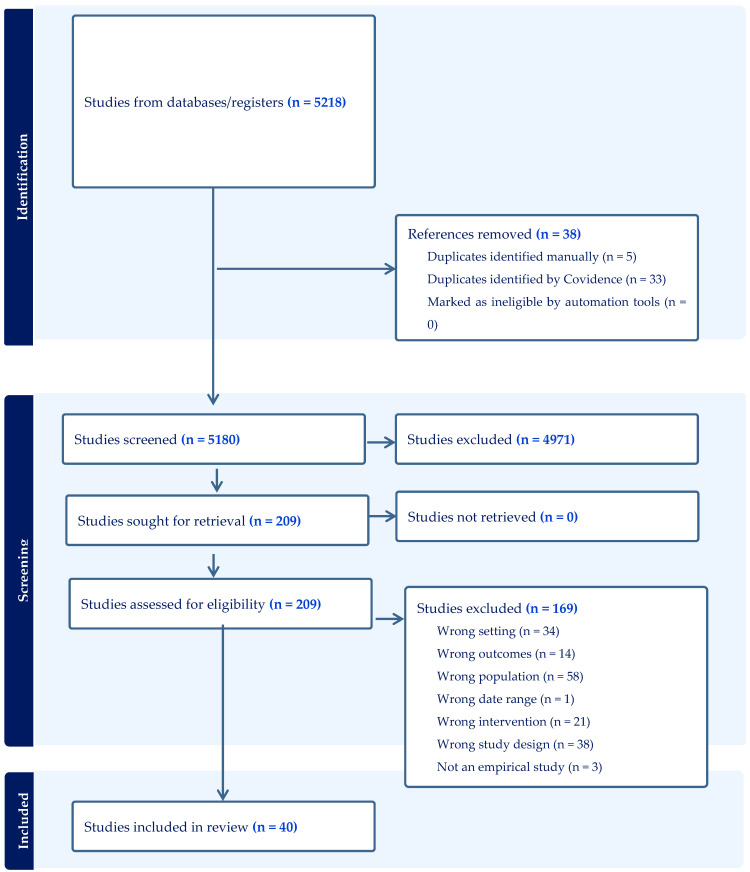
Search results and study selection and inclusion process.

Following the search, all identified citations were collated and uploaded into EndNote (Version 20) and subsequently uploaded to Covidence for removal of duplicates and screening. In the second stage, the articles were evenly divided amongst the authorship team (TG, MD, JM, JB, MS, DE, FP, AH, SH, RMM, AR), with the title and abstract of each article being screened by two reviewers independently and anonymously to confirm whether it met the inclusion criteria. The full text of the remaining items was similarly screened independently and anonymously using the same process. At each screening stage, any conflicting decisions were resolved by a team member who was not involved in the screening of that particular article (JM, AR, or TG). Reasons for exclusion of full-text studies are reported in the scoping review. The search results are presented in a PRISMA flow diagram (see Figure 1).

Data were extracted from papers included in the scoping review by two independent reviewers using a data extraction tool developed by the reviewers following JBI guidelines. Data initially extracted included author(s), publication year, journal, country, study aim, methodology, study design, data collection method, data analysis method, study setting, responder population, sample size, participant age, participant sex or gender, intervention description, types of outcomes, main findings, and other comments. Any disagreements that arose between the reviewers were resolved with an additional reviewer. A total of 40 disagreements in the full text review phase were resolved through an additional reviewer. Cases of disagreements were frequently due to lack of clarity in included studies about the inclusion of risk in the activities investigated or whether settings were natural or man-made environments (e.g., outdoor playgrounds or outdoor areas within the school’s premises).

#### 3.2.7. Synthesis

A descriptive narrative synthesis of the literature was conducted to meet the primary aim of this scoping review, namely, to identify and map the outcomes of nature-based risky play and adventure education for children and youth. This synthesis approach was considered appropriate by the authorship team to effectively manage the heterogeneity of included empirical studies and was guided by the objectives of the study. Extracted data were systematically grouped according to the study’s review objectives.

Given that the final sample included diverse literature types, such as cross-sectional surveys, qualitative studies, randomised controlled trials, and theoretical papers, a formal critical appraisal to assess study quality was not performed. This aligns with best practice for scoping reviews, where the objective is to map the available evidence rather than assess the quality of individual studies for effect estimation, and where a meta-analysis is inappropriate due to significant study heterogeneity (Aromataris et al., 2024).

## 4. Results

The search conducted in August 2025 identified 5218 studies (Figure 1). After the removal of 38 duplicate studies, the remaining 5180 articles were screened. Eligibility screening of titles and abstracts resulted in the further exclusion of 4971 studies for reasons such as ineligible research types, wrong population focus, phenomenon of interest, or study context. Screening of the full texts of the remaining 209 articles led to the further exclusion of 169 studies. No additional studies were identified from reference list screening. A total of 40 studies were included in the final review and extraction.

### 4.1. Study Characteristics

The main characteristics of included studies are presented in Table 1. The studies exhibited considerable diversity in their geographical and methodological characteristics. Geographically, the research was dominated by contributions from the United States (*n* = 15), followed by the United Kingdom (*n* = 6), South Africa (*n* = 4), and Australia (*n* = 4). Studies were also conducted across Europe, including in Norway, Greece, Romania, and Türkiye, as well as in Canada, New Zealand, and Israel, providing a broad international perspective. Methodologically, a qualitative approach was the most common (*n* = 20), utilising designs such as case studies, ethnography, grounded theory, and phenomenography to gain an in-depth understanding of participant experiences. A further 11 studies employed quantitative methods, primarily cross-sectional pre–post surveys, to measure specific outcomes, while nine studies adopted a mixed-methods approach, converging quantitative and qualitative data for a more comprehensive analysis.

Data collection methods were diverse, including self-report questionnaires, interviews, focus groups, observations, video recordings, and accelerometer data. Qualitative analyses typically relied on thematic or template analysis, while quantitative analyses ranged from descriptive statistics and ANOVA to advanced modelling techniques such as latent growth curve modelling and Bayesian approaches.

The settings for these interventions varied, encompassing structured programs like wilderness therapy and outdoor adventure expeditions, as well as integrated educational approaches such as nature-based preschools and regular early childhood education centres with access to outdoor spaces. The participant populations were equally varied, encompassing toddlers, preschoolers, primary and secondary school students, adolescents, and educators. Sample sizes ranged from single-case studies to large-scale surveys with thousands of participants, reflecting both exploratory and confirmatory research traditions.

Age ranges spanned from 17 months to 18 years, with gender distribution generally mixed, although six studies focused exclusively on girls and one study focused on male students only (Bradley & Male, 2017). Notably, six studies targeted vulnerable or underrepresented groups, such as adolescents from low socioeconomic backgrounds (Allan et al., 2025; Slee & Allan, 2019), ethnic minorities (Slee & Allan, 2019), adolescents with emotional, behavioural, or substance-related disorders (Tucker et al., 2016), and children with autism (Bradley & Male, 2017; Friedman et al., 2024; Zachor et al., 2017), signalling an equity focus within the field.

Methodological innovations, such as wearable technology for observational research and the increasing use of longitudinal designs, suggest a maturing evidence base. However, variability in sample sizes and reliance on self-report measures in many studies indicate ongoing challenges in achieving methodological consistency and generalisability.

### 4.2. Study Aims

Study aims can be categorised into three broad areas: psychosocial development and wellbeing; program design, experience, and effectiveness; and early childhood and risk exploration. While most studies fit into only one of these categories, some studies overlapped (see Table 2). Psychosocial development and wellbeing were the most frequently stated aims of the interventions included in this review. Notably, 23 studies focused on areas such as challenge-seeking behaviours, personal and social development, mental health, noncognitive factors, resilience, self-determination, emotional literacy, and basic psychological needs (e.g., autonomy, competence, relatedness), and positive youth development outcomes.

Eleven studies examined the structure, impact, and subjective experience of specific programs, including ways of integrating outdoor education into curriculum, with a focus on the effectiveness of programs for children with autism spectrum disorder (Bradley & Male, 2017; Zachor et al., 2017). These studies evaluated the experiences of pilot programs in an outdoor adventure-based science course (Mackenzie et al., 2018), long-term program impacts (Talley et al., 2023), preferred program components (Down et al., 2023), and motor skill development (Stoica et al., 2025).

Nine studies were dedicated to exploring early childhood and risk. Key variables included risky play behaviours (Deniz & Cevher Kalburan, 2024), educators’ attitudes towards risk-taking (Little, 2022), the effects of unstructured play on creativity and self-confidence (Okur-Berberoglu, 2021), and how toddlers assess and manage risk in a natural environment (Olsen et al., 2025; Tangen et al., 2022).

### 4.3. Interventions

Across the 40 studies reviewed, five main types of interventions were identified, ranging from highly structured OAE to naturally occurring, unstructured play experiences (see Table 3). Half of all studies (*n* = 20) involved organised OAE and expedition programs, typically short term (3–80 days, average 2–3 weeks) and delivered as one-off residential or expedition experiences. These programs were predominantly adult directed and facilitated by trained instructors, emphasising teamwork, leadership, resilience, and personal growth through moderate- to high-risk physical challenges such as climbing, kayaking, hiking, and ropes activities.

A further 11 studies focused on ongoing, curriculum-embedded outdoor learning models such as forest school, bush kindy, or outdoor kindergarten programs. These were long term, child centred, and play based, typically extending across a school term or year. Activities were exploratory and nature immersive, with low to moderate risk exposure through fire building, tree climbing, and tool use, guided by educators acting as facilitators rather than instructors.

Four studies examined schoolyard or playground-based interventions where the physical environment or play opportunities were redesigned or expanded to promote nature and risky play. These ranged from short-term interventions to multiyear extracurricular programs and were primarily educator directed, with low to moderate risk exposure within managed school settings. Only one study investigated an after-school or community-based nature program, such as the Girl Scouts’ outdoor programming, which involved occasional to monthly participation over a year under the guidance of community instructors (Tsikalas & Martin, 2015).

Finally, four studies were observational in design, documenting existing practices or children’s naturally occurring risky play behaviours in outdoor contexts. These studies, often exploratory or ethnographic, provided insights into child-initiated risk negotiation and educator scaffolding without introducing a formal intervention.

### 4.4. Outcomes

The included outcomes from interventions are summarised in Table 4 under the following headings: psychosocial, physical, holistic wellbeing, mental health, academic, and connection to nature. Most included studies focused on the outcomes of nature-based interventions in relation to psychosocial elements, including self-confidence, emotional literacy, resilience, self-efficacy, motivation, autonomy, and overcoming challenge (Allan et al., 2025; Barfield et al., 2021; Barr-Wilson & Roberts, 2016; Bateman & Waters, 2018; Blaine & Akhurst, 2020, 2022a, 2022b; Blum et al., 2025; Bradley & Male, 2017; Brussoni et al., 2017; Card & Burke, 2021; Davidson & Foster, 2024; Down et al., 2023, 2025a; Friedman et al., 2024; Haywood-Bird, 2017; Hinchion et al., 2021; Lazaridis et al., 2023; Mackenzie et al., 2018; Okur-Berberoglu, 2021; Orson et al., 2020; Richmond & Sibthorp, 2019; Richmond et al., 2018; Scogin et al., 2025; Scrutton, 2015; Slee & Allan, 2019; Talley et al., 2023; Tsikalas & Martin, 2015; Whittington & Aspelmeier, 2018; I. R. Williams et al., 2018).

In relation to physical health, three studies investigated the outcomes of physical activity (Barfield et al., 2021; Brussoni et al., 2017; Mackenzie et al., 2018), while others focused on overall health including weight, body image, balance, coordination, and physical fitness (Barfield et al., 2021; Barr-Wilson & Roberts, 2016; Hinchion et al., 2021; Little, 2022; Tucker et al., 2016). Studies also focused on the impact on overall wellbeing from a more holistic point of view (Brusaferro, 2020; Deniz & Cevher Kalburan, 2024; Lazaridis et al., 2023; Rocca, 2022; Tucker et al., 2016; Zygmont & Naidoo, 2018). Outcomes associated with academic skills were highlighted in three papers (Bradley & Male, 2017; Card & Burke, 2021; Scogin et al., 2025), while four studies focused on children’s and adolescents’ connection to the natural world (Brusaferro, 2020; Card & Burke, 2021; Scogin et al., 2025; I. R. Williams et al., 2018).

### 4.5. Main Findings

The findings from the 40 studies were analysed using constant comparative method, and eight themes were identified. The findings from some studies fell into multiple themes, and these have been captured and summarised in Table 5. The following themes are ranked from the most identified to the least: (1) resilience, confidence, and challenge; (2) wellbeing; (3) physical changes and skills; (4) autonomy; (5) nature connectedness; (6) quality play or adventurous activity provision; and (7) participants influence educators. Each theme is discussed in further detail below.

#### 4.5.1. Resilience, Confidence, and Challenge

Twenty-five of the studies highlighted the benefits of building resilience, confidence, and challenging participants. Seven of the studies showed an overall increase in participants’ self-confidence (Barfield et al., 2021; Bateman & Waters, 2018; Bradley & Male, 2017; Card & Burke, 2021; Okur-Berberoglu, 2021; Richmond et al., 2018; Whittington & Aspelmeier, 2018), and two studies specifically identified that girls sought out challenges, and this showed an increase in overall resilience (Barr-Wilson & Roberts, 2016; Tsikalas & Martin, 2015).

A study of OAE demonstrated a significant enhancement of participants’ grit (i.e., perseverance and passion toward long-term goals), mastery, and emotional regulation (Davidson & Foster, 2024). The most valued aspects of OAE were opportunities to develop relationships, and build social connections, self-efficacy, resilience, and a sense of individual empowerment (Down et al., 2023). One study by Bateman and Waters (2018) showed that when adventurous activities are scaffolded to allow for sequential growth, children’s independence, risk-taking, and emotional regulation improved. Another study by I. R. Williams et al. (2018) assessed the impact of an outdoor adventure program on positive adolescent development, and the qualitative data clearly indicated an increase in independence, teamwork, self-awareness, and confidence. In a similar vein, Orson et al. (2020) found that participants enhanced their social–emotional skills during involvement in OAE programs. They concluded that participants learnt through active processes of struggling with challenges, including building perseverance, constructing positive mindsets, and solving social problems. In this study, peers provided critical on-the-spot emotional support that helped facilitate participants to overcome personal hardships and challenges.

A Norwegian study by Tangen et al. (2022) focused on toddlers found that their intervention increased physical competence, confidence, and problem-solving skills. Unstructured outdoor play fostered five core developmental outcomes: observation, exploration, cognitive development, creativity, and self-confidence. Another study discovered that toddlers engaged confidently in play involving heights, balance, speed, and seclusion, particularly while using the tyre tower and rocks (Little, 2022).

#### 4.5.2. Wellbeing

Twenty-one studies noted an improvement in participants’ psychosocial or affective wellbeing. This included increased social and personal connections, social and emotional skills, mental health, self-efficacy, and stronger relationships. An extracurricular program from Greece showed success in promoting adolescents’ basic psychological needs (Lazaridis et al., 2023). Notably, another program provided participants with a greater sense of autonomy and competence compared to typical physical education lessons (Down et al., 2023). A study by Whittington and Aspelmeier (2018) revealed that girls in experiential adventure education programs reported significant improvements in positive peer relationships, while those in traditional camp settings did not.

Interestingly, one study by Brussoni et al. (2017) noted significant decreases in depressed affect (mental health) and antisocial behaviour in participants. Richmond et al. (2018) found that sharing challenges and being away from technology fostered stronger bonds between participants and their educators. Another study found increased self-efficacy in dealing with challenge after completing an intervention program (Richmond & Sibthorp, 2019). Another stated that participants made new friends, felt more connected, and spent less time at home alone after school (Barfield et al., 2021).

A study undertaken in a forest school by Friedman et al. (2024) posited that participants developed stronger peer relationships, resilience, and a sense of belonging and competence. Correspondingly, research by Bradley and Male (2017) identified that when participants with autism spectrum disorder (ASD) were offered unique opportunities in nature, it helped them to talk about friendships, which is a much-needed skill.

#### 4.5.3. Physical Changes and Skills

Fourteen studies noted physical changes in the participants. This included physical skills, fitness, and changes to bodies. In one study of 13 girls, all participants reported that the proGram-positively influenced their body image during and immediately after the course (Barfield et al., 2021). Key positive influences were identified as instructors (as role models), other girls on the course (creating a supportive, nonjudgemental environment), and the natural environment. Participants noted improved physical health, relationship with self, relationships with others, and emotional health.

A Romanian program (Stoica et al., 2025) with young children that included challenging physical activities like slacklining, obstacle courses, rope routes, climbing, and balance games found statistically significant improvements in motor skills among participants. Significant improvements were observed in dynamic balance and general coordination for most groups. Physical fitness scores improved significantly, showing a decrease in the mean score (indicating better fitness) for all groups. Improvements varied by age and gender, with boys and different age groups showing distinct patterns of progress in different skills.

Interestingly, a different study found that participants’ physical activity increased by 121% during the program, yet no significant changes were found in physical activity attitudes or active outdoor identity (Mackenzie et al., 2018). This was likely due to high baseline scores and the short program duration.

#### 4.5.4. Autonomy

An unexpected outcome was the importance of choice, autonomy, and agency, with 10 studies highlighting these outcomes. This feature extends to participants of all age ranges. A study with preschool children from the United States found that children used outdoor play to experiment with and wield individual power and agency (Haywood-Bird, 2017). They noted that key forms of powerful play included physically risky play (e.g., climbing trees), complex imaginative play where children negotiated roles and narratives, and choosing to play alone. The outdoor environment facilitated a fluid give and take of power, allowing children to make choices, lead, follow, or opt out of play without conflict. Another study with toddler participants found that, when offered the opportunity to make choices, they could assess and manage risks through direct (e.g., slowing pace, careful looking) and indirect (e.g., observing peers) strategies (Tangen et al., 2022).

A small study in an urban environment with middle-aged children also found that the participants demonstrated autonomy by navigating and sometimes circumventing adult rules to engage in meaningful risky play (Hinchion et al., 2021). An outdoor adventure educational program also noted that participants developed a sense of individual empowerment and greatly valued autonomy and freedom; however, this sometimes presented a challenge for teachers managing risk (Down et al., 2023). A study by Lazaridis et al. (2023) with older girls acknowledged that their program successfully promoted adolescents’ basic psychological needs, with participants reporting increased feelings of autonomy and freedom of choice.

#### 4.5.5. Nature Connectedness

Nine of the studies highlighted the benefits of connectedness with nature. One study reported that younger participants engaged in a wide range of activities, including gross motor skills, loose materials, and water activities in the natural environment (Olsen et al., 2025). In general, participants developed an ecological identity during nature play, fostering social development, emotional regulation, self-confidence, independence, creativity, and connection to nature. Children developed stronger peer relationships, resilience, and a sense of belonging and competence. A forest school study reported enhanced wellbeing, autonomy, relational safety, environmental connectedness, and opportunities for mastery and reflection (Davidson & Foster, 2024). Another study reports five common themes, namely, (1) relationship and community, (2) perseverance and resiliency, (3) enjoyment and finding beauty in nature, (4) leadership and confidence, and (5) individual growth (Down et al., 2023).

#### 4.5.6. Quality of Play and Adventure Education Provision

Four of the studies highlighted the benefits of quality play provision (Brussoni et al., 2017; Haywood-Bird, 2017; Hinchion et al., 2021; Little, 2022). The open, natural space allowed complex, co-constructed social dramas to emerge organically from the children, free from adult direction. The intervention from Brussoni et al. (2017) dramatically improved the play environment quality, with Seven Cs scores (character, context, connectivity, clarity, change, chance, challenge), rising from 44 to 97 in one centre and from 35 to 125 in the other. By adding natural materials and increasing opportunities for risky play, the intervention increased the number and variety of play affordances, encouraging more play with natural elements. Educators noted richer and more autonomous play, with children engaging in more independent and prosocial behaviours, demonstrating greater creativity, self-regulation, confidence, and lower levels of stress and boredom. A study with preschool children from the United States found that children used outdoor play to experiment with and wield individual power and agency (Haywood-Bird, 2017). Key forms of powerful play included physically risky play (e.g., climbing trees), complex imaginative play where the participants negotiated roles and narratives, and even choosing to play alone. Quality play provision also allowed participants to increase their use of natural loose and fixed elements and used different and more areas of the play space.

#### 4.5.7. Participants Influence Educators

Three of the studies identified that the child and youth participants were able to influence the attitudes and behaviours of educators (Little, 2022; Slee & Allan, 2019; Tangen et al., 2022). Educators developed greater trust in children’s abilities and became more comfortable with risk. One study found that teachers emphasised the need for children’s freedom to plan and explore their emerging experiences (Little, 2022). Reflection by the educators on their personal attitudes and tolerance for risk-taking led to a marked shift towards risk-positive pedagogies, which in turn reinforced children’s agency and independence.

## 5. Discussion

This scoping review set out to identify the outcomes of nature-based risky play and adventurous activities for children and adolescents up to 18 years of age. Specifically, we wanted to interrogate the literature surrounding the physical, mental health, and psychosocial outcomes of outdoor risky play or adventure education for childhood and adolescence. The near-universal positive findings across all 40 studies, regardless of methodological approach, geographical location, or intervention type, present compelling evidence that nature-based risky play and adventurous outdoor activities generate multifaceted developmental benefits for children and adolescents. What distinguishes this evidence base is not merely the consistency of positive outcomes but their interconnected nature. The reviewed literature demonstrates that nature-based risk-taking serves multiple functions simultaneously rather than a singular function, such as socioemotional skills, anxiety reduction, or physical fitness. These findings align with other scholarly works spanning many decades in the field (Beames et al., 2012; Hattie et al., 1997; Mutz & Müller, 2016).

Emerging research in the area of neuroscience indicates that early experiences in natural settings help shape children’s brain development and set strong foundations for adolescence and lifelong health and wellbeing (Eager et al., 2025; T. Gray et al., 2025a). Holistic benefits cover psychosocial wellbeing, physical development, cognitive function, social competence, and emotional regulation, which is also in accord with similar studies (T. Gray & Martin, 2012; Kahn & Kellert, 2002; Mann et al., 2022). The evidence is clear; children and adolescents need healthy risk-taking in outdoor environments to flourish and thrive (D. D. Williams, 2022). These enrichment opportunities have been shown to transfer into adulthood as the benefits ripple throughout society (Capaldi et al., 2014; T. Gray et al., 2025b).

The fact that structured, adult-led outdoor adventure programs (50% of studies) produced similarly positive outcomes to child-initiated, unstructured play experiences (10% of studies) suggests that the core mechanism remains consistent: meaningful engagement with manageable challenge in natural environments. This pattern is reinforced by Mann et al. (2022), who clearly indicated that effective risky play and adventure education opportunities can be implemented across diverse contexts, ranging from wilderness expeditions to redesigned schoolyards. In essence, multiple pathways exist to achieve developmental benefits, transcending specific program models.

An unexpected yet consistent finding emerged across 14 studies concerning the centrality of child autonomy, choice, and self-directed decision-making in generating developmental benefits. This theme appeared with equal prominence across early childhood, middle childhood, and adolescent samples. The evidence suggests that opportunities for authentic agency constitute a fundamental component of effective risk-taking experiences rather than a peripheral consideration. Children as young as toddlers demonstrated a capacity for nuanced risk assessment when afforded choice, while adolescents explicitly valued autonomy and freedom despite the management challenges this created for educators. This finding aligns with self-determination theory (Ryan & Deci, 2000) and extends the existing literature by suggesting that the benefits of nature-based risky play may be substantially mediated by the satisfaction of autonomy needs, working alongside competence and relatedness.

Several studies have documented a particularly important pattern: children’s demonstrated competence in managing risks encouraged educators to expand permissions for risk-taking, which in turn enhanced children’s confidence and skill development. This bidirectional feedback loop suggests that authentic agency functions as both a feature of optimal risky play implementation and potentially as a mechanism through which developmental gains are realised.

Intervention studies targeting educator and parental attitudes yielded mixed results. Professional development workshops increased confidence but showed insufficient impact on fundamental beliefs about risk. Translating evidence into practice requires attention not only to educator knowledge and skills, but also to the deeper cultural narratives and institutional structures that shape how communities view risk.

Additionally, the findings from studies that uncovered participants with ASD experienced unique social and relational benefits from risky outdoor play carry important implications. Combined with evidence that children without risky play experience more serious injuries due to poor risk assessment skills (Dickson et al., 2008), this points to an overlooked equity dimension in risk-aversion discourse. This begs the question: If we continue to deny children and adolescents the opportunity for healthy risk-taking, does this constitute a form of developmental harm?

A critical challenge emerged throughout this review that has substantial implications for both research and practice communication. Across the 40 studies, researchers employed inconsistent and overlapping terminology to describe what appear to be similar developmental outcomes. Terms such as resilience, confidence, self-efficacy, grit, emotional literacy, and mastery were often used interchangeably to describe comparable phenomena, while frameworks incorporating these constructs varied from study to study. Additionally, broader vocabularies competed across the literature, including “21st century skills,” “soft skills,” “noncognitive factors,” “character development,” and “personal and social development,” each carrying different theoretical assumptions and measurement approaches. This terminological fragmentation creates significant obstacles for evidence synthesis and practice translation. Future research would benefit substantially from establishing consensus on outcome definitions and measurement frameworks. Such standardisation would not diminish recognition of risky play’s multifaceted benefits but would strengthen the field’s capacity to document, compare, and communicate these benefits with precision and coherence.

Indeed, in addition to issues of language, the methodological quality of the included studies could be questionable due to a lack of reporting on statistical significance, sampling methods, validity and reliability of outcome measures, and attrition or dropout rates not reported, and some studies may have employed suboptimal research designs. Best practice frameworks for assuring methodological rigour, such as Cochrane’s Risk of Bias Tool, the CONSORT (Consolidated Standards of Reporting Trials), or STROBE (Strengthening the Reporting of Observational Studies in Epidemiology) for quantitative studies or the use of critical appraisal checklists for qualitative studies, such as the ones available from JBI, would allow for more consistency in reporting and assurance of quality. Variability in sample sizes and reliance on self-report measures in many studies also indicate ongoing challenges in achieving methodological consistency and generalisability. Additionally, some studies did not explicitly state whether the children or adolescents included in the studies had any pre-existing medical conditions, which may have impacted the outcomes. As such, it is difficult to establish generalisability of the findings when there are widespread variations in study designs and the methodology lacks a robust and rigorous nature.

### 5.1. Limitations

While this scoping review used a robust, comprehensive, and widely utilised methodology, there are still some notable limitations. The evidence base raised many concerns regarding the ambiguity and consistency of the language used in the reported outcomes. Ostensibly, interpretation was then left open to the researchers’ interpretations of the nuanced language, which may bring a personal bias. The ambiguity could be addressed by clearer reporting, and ideally, future studies should ensure consistency in outcome nomenclature.

### 5.2. Implications for Future Research and Practice

The evidence supporting nature-based risky play and adventure education as enrichment opportunities for children and adolescents is growing in popularity, yet implementation remains inconsistent and fragmented. Why, then, does implementation lag so far behind the evidence? Clearly, it is not a knowledge problem. Researchers have documented the benefits, educators are increasingly recognising their importance, and policymakers have access to this evidence. The gap appears to reflect institutional inertia, societal anxiety about childhood risk, and liability frameworks that accentuate harm prevention. Addressing this gap would benefit from fundamental reconsideration of liability structures, curriculum frameworks, and how we conceptualise childhood and adolescence as periods requiring meaningful challenge and exploration in addition to appropriate protection.

Several implications for researchers and practitioners warrant consideration.

For researchers: Establishing standardised outcome measurement frameworks would strengthen the field considerably. Future studies should adopt consistent terminology and definitions of constructs such as resilience, self-efficacy, and emotional regulation. Longitudinal designs tracking sustained effects would enhance understanding, as would studies expanding beyond predominantly Western contexts to include culturally diverse settings and vulnerable populations including children from low socioeconomic backgrounds, ethnic minorities, and children with disabilities. Comparative research examining intervention effectiveness across age groups and contexts could clarify optimal implementation strategies.

For practitioners: Educational policymakers should consider positive risk-taking as central to child and adolescent development, rather than peripheral enrichment. Curriculum frameworks would benefit from incorporating nature-based risk-taking opportunities across school years. Preservice teacher education programs would be strengthened through expanded focus on designing and facilitating risky play or adventurous activities. Professional development for in-service teachers could address the cultural narratives and institutional beliefs shaping educator responses to risk. Institutional policies emphasising blanket risk minimisation might be reconsidered to permit informed risk-taking aligned with developmental research. Environmental design in schools and communities could prioritise natural elements and affording varied risky play opportunities. Importantly, practitioners could consider creating structured opportunities for children and adolescents to exercise genuine choice and independent decision-making within these contexts, as the evidence suggests that autonomy is important for positive outcomes.

## 6. Conclusions

Evidence-based research is amassing to indicate that early experiences shape children’s brain development and set strong foundations for adolescence and lifelong health and wellbeing. This scoping review of 40 empirical studies indicates that nature-based risky play and adventurous outdoor activities produce wide-ranging developmental benefits for children and adolescents across physical, cognitive, emotional, and social domains. The uniformity of positive findings across diverse methodologies and contexts provides a substantial evidence base for meaningful policy and practice improvements.

What distinguishes this evidence base is the consistency with which studies suggest that authentic agency and autonomy function as important mechanisms through which benefits may be realised. Children and adolescents who exercise genuine choice in assessing and managing risk develop not only immediate competencies but also increased confidence in their capacity to handle life’s challenges. Yet, current educational systems often limit children’s access to these experiences and paradoxically may contribute to new challenges: potentially compromised benefit–risk assessment capacities, diminished emotional resilience, and declining mental wellbeing.

Providing all children and adolescents with genuine opportunities for nature-based risk-taking and authentic decision-making is not optional; it is fundamental to equitable, ethical education. The evidence provides a strong foundation for transformational change. What remains is the institutional and societal commitment and willpower to translate these findings into practices that support a child’s and adolescent’s holistic development. Future research should prioritise longitudinal studies, cross-cultural investigations, and equity-focused approaches that address disparities in access to positive risk-taking.

## Figures and Tables

**Table 1:** Main Characteristics of Included Studies.

Author(s) Publication Year	Country	Methodology	Study Design	Data Collection Method	Data Analysis Method	Study Setting	Responder Population	Sample Size	Participants Age Range	Participants Gender
Allan et al. (2025)	United Kingdom	Quantitative	Repeated measures (pre, post, and 1-month follow-up)	Survey	Paired *t* tests, repeated measures ANOVA, correlation analyses, and multiple linear regressions	Outdoor adventure residential centres	Diverse adolescents, including those from disadvantaged backgrounds and with special educational needs and disabilities	622 participants (main intervention group for pre/post), 301 at 1-month follow-up	16–17 years	Mixed
Barfield et al. (2021)	United States	Qualitative	Descriptive qualitative	Students: focus group and art-based methods Parents: focus group	Content analysis	Rural after-school program	Students and parents	16 students and 6 parents	Mean age 14 years	Mixed
Barr-Wilson and Roberts (2016)	United States	Mixed methods	Retrospective phenomenological inquiry	Written questionnaires and focus groups	Thematic analysis	A nonprofit outdoor adventure organisation	High-school-aged alumnae of the organisation	13	13–16 years	Female
Bateman and Waters (2018)	New Zealand	Qualitative	Case study	Video and audio recordings, field notes, written reflections	Conversation analysis combined with the Leuven Wellbeing Scale for observational assessment	Crèche with access to a local bush area	Early childhood educators and preschool children	Not stated	4 years	Mixed
Blaine and Akhurst (2020)	South Africa	Mixed methods	Quasi-experimental with qualitative inquiry	Quantitative: self-report questionnaires at three points in time: pre, post, and 4 months later Qualitative: focus groups, semistructured interviews, post-evaluation surveys, letters to principals	Quantitative: repeated measures ANOVA, effect sizes Qualitative: template analysis	Independent single-sex schools	Students	144	Mean age 16.5 years	Mixed
Blaine and Akhurst (2022a)	South Africa	Qualitative	Descriptive qualitative, longitudinal	Semistructured focus group interviews, one-on-one interviews, post-journey surveys, and analysis of learners’ letters to their principals	Template analysis	Independent single-sex schools	Students	144 participants for surveys/letters 20 for in-depth focus groups	Grade 10, average age 16.5 years	Mixed
Blaine and Akhurst (2022b)	South Africa	Quantitative	Cross-sectional	Self-report questionnaires	Repeated-measures ANOVA, pairwise comparisons, effect sizes	Independent single-sex high schools	Year 10 students	144	16 years	Mixed
Blum et al. (2025)	United States	Quantitative	Cross-sectional	Pre–post self-reported surveys	MANOVA	Tall ship training	Youth	358	8–17 years	Mixed
Bradley and Male (2017)	United Kingdom	Qualitative	Case study	Children: video-stimulated recall, semistructured interviews with visual prompts, and drawing activities Adults: semistructured interviews	Thematic analysis	An inner-city special school	Young children with a primary diagnosis of ASD and additional severe learning difficulties, their parents, and their teaching assistants	4 children, 3 mothers, and 2 teaching assistants	6–8 years	Male
Brusaferro (2020)	United States	Qualitative	Case study	Unstructured child interviews, daily “favourite part of the day” conferencing, parent anecdote circles, biographical questionnaires, teacher debrief sessions, and curriculum documentation	Thematic qualitative coding and narrative analysis	Forest preschool	Children, mothers, teachers	19 children, 17 mothers, 3 teachers	3–5 years	Mixed
Brussoni et al. (2017)	Canada	Mixed methods	Convergent mixed methods repeated measures design	Questionnaires (completed by early childhood educators), accelerometers, video-recorded play observations, spatial behaviour maps, and focus groups with educators	Quantitative: Wilcoxon signed-rank tests, generalized linear mixed effects models (GLMM) Qualitative: thematic analysis of focus groups and play observations	Childcare centres	Children, early childhood educators	45 children and 16 educators	2–5 years	Mixed
Card and Burke (2021)	Canada	Qualitative	Narrative single-case study	Free-play interviews, observations, field notes, artifacts, photographs, audio and video recordings	Not explicitly stated, but consistent with qualitative thematic analysis of narrative data	Nature school	Families (including the children) and the school’s founder/director	4 families (specific number of children not provided)	Not explicitly stated, but the context is Kindergarten	Mixed
Davidson and Foster (2024)	United States	Quantitative	Cross-sectional	Pre- and post-course surveys	Bayesian latent growth modelling and latent change models	OAE programs	Adolescents	350	14–18	Mixed
Deniz and Cevher Kalburan (2024)	Turkey and Germany	Qualitative	Case study	Researcher-designed observation forms, frequency records, researcher notes, and photographs	Qualitative content analysis	Nature-based preschools	Children	58	3–6 years	Mixed
Down et al. (2025a)	Australia	Qualitative	Descriptive qualitative, cross-sectional	Semistructured focus groups with adolescents and key informant interviews with OAE teachers	Template thematic analysis	Independent schools	Adolescent students and OAE teachers	29 adolescents and 4 key informant teachers	15–16 years	Mixed
Down et al. (2023)	Australia	Qualitative	Descriptive qualitative	Semistructured student focus groups and semistructured key informant interviews	Template thematic analysis	Independent schools	Adolescent students and OAE teachers	29 students and 4 key informant teachers	15–16 years	Mixed
Friedman et al. (2024)	United Kingdom	Qualitative	Ethnographic case study	Participant observation, semistructured interviews with parent and children	Deductive reflexive thematic analysis	A forest school program within the grounds of a specialist school for autistic children	Autistic children and their parents	25 children observed, 10 parents interviewed, and 9 children interviewed	Mean age = 9.8 years	Mixed
Haywood-Bird (2017)	United States	Qualitative	Ethnography	Participant observation, handwritten field notes, and informal interactions with children	Thematic analysis	A private, nonprofit, Waldorf-inspired preschool	Preschoolers	25	2–5 years	Mixed
Hinchion et al. (2021)	Ireland	Qualitative	Ethnography	Focus groups, drawings, photography, child-led tours, map-making, and observations	Thematic analysis	Small rural town	Children	10	6–8 years	Mixed
Lazaridis et al. (2023)	Greece	Qualitative	Descriptive qualitative, longitudinal	Semistructured interviews	Thematic analysis	A secondary school	Adolescent students	12	12–14 years	Mixed
Little (2022)	Australia	Mixed methods	Case study	Quantitative survey tools and qualitative observation and interviews	Deductive qualitative coding Descriptive statistics and correlations	Early childhood centre	Children (videos), educators (interviews)	20	Early childhood	Mixed
Mackenzie et al. (2018)	United States	Quantitative	Cross-sectional	Self-report survey and pedometer	Descriptive statistics and ANOVA	Residential science school near a lake, state park, and ski area	High school students	22	Mean age 15.7 years	Mixed
Okur-Berberoglu (2021)	New Zealand	Qualitative	Case study	Nonparticipant observations, photographs, and observation notes from the researcher (the child’s mother) and an early childhood education teacher	Content analysis	A kindergarten-run Bush Explorer program	One preschool child	1	4 years	Female
Olsen et al. (2025)	Norway	Mixed methods	Observational study	Video recordings from head- or chest-mounted GoPro cameras	Quantitative coding of video data (second-by-second) using Observer XT software for time allocation, supplemented by qualitative descriptions of activities	Natural environments close to early childhood education and care centre	Children	7	17–25 months	Mixed
Orson et al. (2020)	United States	Qualitative	Grounded theory	Group interviews	Iterative grounded theory analysis	Outward Bound expeditions in wilderness environments	Adolescents	32	14–18 years	Mixed
Richmond and Sibthorp (2019)	United States	Mixed methods	Cross-sectional	Quantitative: pre and post self-report questionnaires Qualitative: semistructured interviews	Quantitative: multilevel models Qualitative: open and axial coding and thematic analysis	College access program	Adolescents	165	14–16 years	Mixed
Richmond et al. (2018)	United States	Qualitative	Grounded theory	Semistructured interviews	Qualitative analysis using open, focused, and axial coding	An independent all-girls school	Adolescent students and adult faculty	31 students and 8 faculty members	13–18 years	Female
Rocca (2022)	United Kingdom	Qualitative	Grounded theory	Semistructured photo-elicitation interviews	Grounded theory	Forest school	Children, parents, facilitators	5 children, 5 parents, and 5 facilitators	5–12 years	Mixed
Scogin et al. (2025)	United States	Mixed methods	Convergent mixed-methods research design	Quantitative: instruments—observation-based assessment (TS GOLD), literacy test (DIBELS), survey (Connection to Nature Index) Qualitative: focus group and electronic questionnaire	Quantitative: Wilcoxon signed-rank tests, frequency distributions, descriptive statistics Qualitative: inductive, constant comparative method for thematic analysis	Preschool program	Children and caregivers	69 children and 15 caregivers	4–5 years	Mixed
Scrutton (2015)	United Kingdom	Quantitative	Cross-sectional	Self-report questionnaire	Nonparametric statistical tests, effect size calculation, reliability analysis, and factor analysis	Primary schools and residential outdoor centres	Students	Experimental group: 360 pupils Control group: 115 pupils	10–12 years	Mixed
Slee and Allan (2019)	United Kingdom	Mixed methods	Comparative intervention study with a quasi-experimental quantitative component and a qualitative component	Questionnaire, semistructured interviews, information group discussions	Quantitative: descriptive and parametric statistical analysis Qualitative: thematic analysis	Secondary schools and an outdoor adventure centre	School children preparing to transition to high school from ethnic monitories and low socioeconomic groups	100	Mean age 11 years	Mixed
Stoica et al. (2025)	Romania	Quantitative	Cross-sectional	Direct observation and recording of scores on standardised physical tests	Descriptive and inferential statistical analysis	School camp	Students	228	11–14 years	Mixed
Talley et al. (2023)	United States	Qualitative	Narrative enquiry, case study	Photo-elicitation in focus group interviews	Thematic analysis	A private middle school	8th grade students	24	8th graders	Female
Tangen et al. (2022)	Norway	Qualitative	Descriptive qualitative	Video observation using head-mounted GoPro cameras	Inductive thematic analysis	Varied natural environments near early childhood education and care centres	Children	7	17–25 months	Mixed
Tsikalas and Martin (2015)	United States	Quantitative	Cross-sectional	Online survey	Descriptive statistics, correlations, hierarchical regression analyses, and thematic coding of open-ended comments	Girl Scouts of the USA nationwide	Girls Scouts (juniors and cadettes)	2862	8–14 years	Female
Tucker et al. (2016)	United States	Quantitative	Cross-sectional	Physical health measures Mental health measures: self-reporting questionnaire	Statistical analysis, bivariate correlations	Nomadic wilderness therapy program	Adolescents with emotional, behavioural, or substance-related disorders	516	13–18 years	Mixed
Whittington and Aspelmeier (2018)	United States	Quantitative	Cross-sectional	Self-report questionnaire (pre and post)	Mixed model ANOVA	All-girls programs (camps and organisations)	Adolescent girls	711	10–17 years	Female
I. R. Williams et al. (2018)	Australia	Mixed methods	Quasi-experimental controlled crossover trial	Online surveys and journals/reflections	Quantitative: latent growth curve modelling Qualitative: thematic analysis	Wilderness setting	Year 9 secondary school students	335 participants (219 in the camp group, 116 in the control group)	14–16 years	Mixed
Zachor et al. (2017)	Israel	Quantitative	Controlled study (nonrandomised, with an intervention and a control group)	Surveys × 3 completed by parents and teachers	Statistical analysis including ANOVA and MANOVA with repeated measures	ASD special education kindergartens and urban parks	Children with ASD enrolled in special education kindergartens	51 participants (intervention group: *n* = 30; control group: *n* = 21)	3–7 years	Mixed
Zygmont and Naidoo (2018)	South Africa	Qualitative	Phenomenography	Semistructured interviews	Phenomenographic analysis	School-based wilderness adventure program	Adolescents	37	14–15 years	Mixed

Note. ANOVA = Analysis of Variance; MANOVA = Multivariate Analysis of Variance; ASD = Autism Spectrum Disorder; OAE = Outdoor Adventure Education; TS GOLD = Teaching Strategies GOLD; DIBELS = Dynamic Indicators of Basic Early Literacy Skills.

**Table 2:** Study Aims of Included Studies.

Category	Author	Aim
Psychosocial development and wellbeing	(Allan et al., 2025)	To report the immediate and enduring impact of a bespoke 5-day outdoor adventure education residential program upon the psychological resilience, wellbeing, and wider skill development of young people.
	(Barfield et al., 2021)	To understand the influence of a rural outdoor afterschool program on adolescent activity and health-related behaviours.
	(Barr-Wilson & Roberts, 2016)	To explore the influence of an outdoor adventure program on body image in adolescent girls and to understand how the participants define “healthy living.”
	(Bateman & Waters, 2018)	To examine how teacher–child interactions during everyday outdoor play support children’s risk-taking, resilience, and wellbeing.
	(Blaine & Akhurst, 2020)	To investigate the efficacy of the “Journey” outdoor adventure education program in enhancing life effectiveness, emotional literacy, and resilience in high school learners and to explore learners’ perceived costs and benefits.
	(Blaine & Akhurst, 2022a)	To explore the psychosocial outcomes and perceived value of a school-based outdoor adventure education program (“Journey”) for adolescents in South Africa.
	(Blaine & Akhurst, 2022b)	To examine whether participation in the Journey outdoor adventure education program improved adolescents’ life effectiveness skills, emotional literacy, and resilience and to explore learners’ perceived costs and benefits of participation.
	(Blum et al., 2025)	To determine changes in Positive Youth Development (PYD) assets reported by participants.
	(Brusaferro, 2020)	To identify and describe the indicators of ecological identity development in children at a forest preschool through nature play.
	(Davidson & Foster, 2024)	To examine the impact of outdoor adventure education on developing grit and resilience in adolescents and to explore how antecedent variables (age, gender, etc.) influence these outcomes.
	(Down et al., 2025a)	To explore the attitudes and opinions of outdoor adventure education teachers and adolescent students regarding the psychosocial outcomes of outdoor adventure education programs, specifically in relation to social connection, belonging, responsibility, challenge, and success.
	(Haywood-Bird, 2017)	To discover how preschool-aged children use play to develop powerful agency in the outdoors and to document their experiences.
	(Lazaridis et al., 2023)	To explore the perceptions and experiences of adolescents and assess their basic psychological needs (autonomy, competence, relatedness) after participating in a 2-year outdoor adventure education program.
	(Okur-Berberoglu, 2021)	To determine the effects of unstructured outdoor play on a young child’s observation, exploration, cognitive development, creativity, and self-confidence.
	(Orson et al., 2020)	To explore how adolescents experience and learn from challenges in outdoor adventure education programs, with a specific focus on the role of peer support and group culture in shaping social–emotional learning (SEL) outcomes.
	(Richmond et al., 2018)	To understand how a shared outdoor adventure education program within a school partnership contributes to the development of noncognitive factors in adolescent students and how these outcomes support success back in school.
	(Richmond & Sibthorp, 2019)	To explore the relation of outdoor adventure education experiences to the development of noncognitive factors among adolescents in a college access program.
	(Rocca, 2022)	To investigate the impact of Forest School on children’s wellbeing and explore potential psychological processes involved, from the perspective of children, parents, and facilitators.
	(Scogin et al., 2025)	To investigate a short-term, nature-based summer preschool program to determine how the program affected children’s social–emotional skills, academic growth, and connections to nature.
	(Scrutton, 2015)	To provide statistically determined evidence on the benefits of outdoor adventure education for personal and social development in Scottish school children.
	(Slee & Allan, 2019)	To investigate the efficacy of three contrasting induction programs for facilitating improvements in children’s psychological wellbeing and self-determination during their transition into secondary school.
	(Tsikalas & Martin, 2015)	To explore how the breadth and intensity of girls’ exposure to outdoor activities in Girl Scouts contributes to their challenge-seeking behaviours and beliefs.
	(Tucker et al., 2016)	To examine changes in both body composition and mental health outcomes among adolescents participating in a wilderness therapy program.
	(Whittington & Aspelmeier, 2018)	To analyse whether girls’ levels of resilience increased after participation in various programs and to compare outcomes across different program types.
Program design, experience, and effectiveness	(Bradley & Male, 2017)	To explore the views of young children with autism spectrum disorder, their parents, and educational professionals about their Forest School experience.
	(Card & Burke, 2021)	To understand the outdoor kindergarten concept and how it provides insight for current kindergarten curriculum, specifically examining how play-based literacy objectives can be met through an outdoor learning classroom.
	(Down et al., 2023)	to gauge the perceptions of adolescents and outdoor adventure education teachers on their preferred program components to improve adolescent wellbeing during a secondary school outdoor adventure education program.
	(Friedman et al., 2024)	to explore the participatory experiences of autistic children at forest school and its impact on their wellbeing, framed by self-determination theory.
	(Mackenzie et al., 2018)	To evaluate student experiences in a pilot outdoor adventure-based science course compared to their normal school settings, focusing on engagement, intrinsic motivation, and physical activity.
	(Scogin et al., 2025)	To investigate a short-term, nature-based summer preschool program to determine how the program affected children’s social–emotional skills, academic growth, and connections to nature.
	(Stoica et al., 2025)	To examine the impact of an adventure education program on the motor skills, coordination, and physical endurance of students aged 11 to 14.
	(Talley et al., 2023)	To examine the impacts of long-term outdoor adventure programming on adolescent girls by exploring their individual and collective experiences.
	(Whittington & Aspelmeier, 2018)	To evaluate the effectiveness of a purpose-designed outdoor adventure program in promoting positive development and reducing vulnerability amongst Year 9 students, both immediately post-program and at 6-month follow-up.
	(Zachor et al., 2017)	To examine the effectiveness of an outdoor adventure program as an additional intervention for young children with autism spectrum disorder.
	(Zygmont & Naidoo, 2018)	Investigate the different ways adolescents experienced a wilderness adventure program and identify aspects of the program that were critical to variation in program outcomes.
Early childhood and risk exploration	(Brusaferro, 2020)	To identify and describe the indicators of ecological identity development in children at a forest preschool through nature play.
	(Brussoni et al., 2017)	To examine the effects of an intervention to increase opportunities for nature and risky play in the outdoor play environments of two childcare centres on children’s play, social behaviours, psychological wellbeing, and physical activity.
	(Deniz & Cevher Kalburan, 2024)	To explore potential and actualized affordances for risky play in two preschool settings.
	(Haywood-Bird, 2017)	To discover how preschool-aged children use play to develop powerful agency in the outdoors and to document their experiences.
	(Hinchion et al., 2021)	To explore children’s subjective experiences and the meaning of risky play in their everyday contexts and environments.
	(Little, 2022)	To examine how the redesign of an outdoor environment influenced toddlers’ risky play behaviours and educators’ attitudes towards risk-taking in early childhood education and care settings.
	(Okur-Berberoglu, 2021)	To determine the effects of unstructured outdoor play on a young child’s observation, exploration, cognitive development, creativity, and self-confidence.
	(Olsen et al., 2025)	To investigate how toddlers use a varied natural environment when allowed to explore freely.
	(Tangen et al., 2022)	To investigate how toddlers assess and manage risk in free exploration in a varied natural environment.

**Table 3:** Intervention Types of Included Studies.

Intervention Type (*n*)	Intervention Characteristics	Studies
Organised outdoor adventure education and expeditions (20)	Duration: Short term (3–80 days), average 2–3 wks, usually once off Main activity: structured, time-bounded residential or expeditionary adventure programs (backpacking, kayaking, ropes, etc.) Purpose/outcome focus: often linked to personal development, physical and mental health, teamwork, or leadership; most correspond to Outward Bound/outdoor adventure education frameworks Risk level: level of activity risk: moderate–high risk exposure, (controlled exposure to height, speed, natural elements, physical challenge) Directed by: trained instructors or educators	(Allan et al., 2025; Barr-Wilson & Roberts, 2016; Blaine & Akhurst, 2020, 2022a, 2022b; Blum et al., 2025; Davidson & Foster, 2024; Down et al., 2023; Down et al., 2025a; Mackenzie et al., 2018; Orson et al., 2020; Richmond et al., 2018; Richmond & Sibthorp, 2019; Scrutton, 2015; Slee & Allan, 2019; Talley et al., 2023; Tucker et al., 2016; Whittington & Aspelmeier, 2018; I. R. Williams et al., 2018; Zygmont & Naidoo, 2018)
Ongoing, curriculum-embedded outdoor learning (Forest School, Outdoor Kindergarten, Bush Kindy, nature preschool) (11)	Duration: weeks—academic year Main activity: play and inquiry in woodland/natural settings (fire-building, den building, climbing, nature crafts) Purpose/outcome focus: social, emotional, cognitive, and academic development through experiential, child-led play Risk level: low–moderate (natural elements, tools, climbing) Directed by: educators/facilitators supporting child-led learning	(Barfield et al., 2021; Bateman & Waters, 2018; Bradley & Male, 2017; Brusaferro, 2020; Card & Burke, 2021; Deniz & Cevher Kalburan, 2024; Friedman et al., 2024; Haywood-Bird, 2017; Okur-Berberoglu, 2021; Rocca, 2022; Scogin et al., 2025)
Schoolyard/playground interventions and extracurricular outdoor programs (4)	Duration: one-off—multiterm (weeks–years) Main activity: redesigned play spaces, extracurricular physical or creative play, outdoor trips Purpose/outcome focus: encourage nature and risky play, physical activity, creativity, social skills Risk level: low–moderate (managed school environment) Directed by: educators	(Brussoni et al., 2017; Lazaridis et al., 2023; Stoica et al., 2025; Zachor et al., 2017)
After-school/community nature-based programs (1)	Duration: occasional to monthly over a year Main activity: outdoor programming across various contexts (casual, service, camping Purpose/outcome focus: Girl Guides Risk level: low to moderate (structured physical challenge) Directed by: community instructors/program leaders	(Tsikalas & Martin, 2015)
Observational/existing practice (nonintervention) studies (4)	Duration: varies (single session—1 year) Main activity: naturally occurring risky play episodes (bush walks, free exploration, natural play) Purpose/outcome focus: descriptive/analytical—understanding children’s self-directed risk-taking, educator responses, or cross-cultural comparisons Risk level: varies (child initiated) Directed by: children (observed by researchers/educators)	(Hinchion et al., 2021; Little, 2022; Tangen et al., 2022; Olsen et al., 2025)

**Table 4:** Outcomes Investigated in Included Studies.

Outcome Type (*n*)	Sample Areas	Studies
Psychosocial (30)	Self-confidence Emotional literacy Resilience Self-efficacy Body image Motivation Autonomy Overcoming challenge	(Allan et al., 2025; Barfield et al., 2021; Barr-Wilson & Roberts, 2016; Bateman & Waters, 2018; Blaine & Akhurst, 2020, 2022a, 2022b; Blum et al., 2025; Bradley & Male, 2017; Brussoni et al., 2017; Card & Burke, 2021; Davidson & Foster, 2024; Down et al., 2023, 2025a; Friedman et al., 2024; Haywood-Bird, 2017; Hinchion et al., 2021; Lazaridis et al., 2023; Mackenzie et al., 2018; Okur-Berberoglu, 2021; Orson et al., 2020; Richmond et al., 2018; Richmond & Sibthorp, 2019; Scogin et al., 2025; Scrutton, 2015; Slee & Allan, 2019; Talley et al., 2023; Tsikalas & Martin, 2015; Whittington & Aspelmeier, 2018; I. R. Williams et al., 2018)
Physical (7)	Physical activity Body Mass Index Balance Coordination Physical fitness	(Barfield et al., 2021; Barr-Wilson & Roberts, 2016; Brussoni et al., 2017; Hinchion et al., 2021; Little, 2022; Mackenzie et al., 2018; Tucker et al., 2016)
Holistic development (6)	Mental health Holistic child development Overall wellbeing Mental health functioning Positive youth development	(Brusaferro, 2020; Deniz & Cevher Kalburan, 2024; Lazaridis et al., 2023; Rocca, 2022; Tucker et al., 2016; Zygmont & Naidoo, 2018)
Academic (3)	Practical and academic learning outcomes Academic achievement Development of critical thinking Problem-solving Creativity Academic readiness	(Bradley & Male, 2017; Card & Burke, 2021; Scogin et al., 2025)
Connection to nature (4)	Development of an ecological identity Environmental sensitivity Connection to nature Nature relatedness	(Brusaferro, 2020; Card & Burke, 2021; Scogin et al., 2025; I. R. Williams et al., 2018)

**Table 5:** Main Findings of Included Studies.

Category	Author	Findings
Resilience, confidence and challenge (25)	(Allan et al., 2025)	Significant increases in resilience (36.33%) and well-being (23.12%) from pre- to post-test. These gains were largely retained at the one-month follow-up. The most powerful predictors of positive change were: being inspired by the countryside, solving one’s own problems, and having freedom of choice. Participants reported developing a broad array of 21st Century Skills.
	(Barfield et al., 2021)	Increasing Health-Related Competencies: Students increased their physical activity, improved their sleep, perceived less stress, and reported changes in dietary habits and electronic use. Increasing Social Relatedness: Students made new friends, felt more connected, and spent less time home alone after school. Increasing Autonomy and Intrinsic Motivation: Students recognized their emerging capabilities, and their increased confidence stimulated more action-oriented behaviour. Parent-perceived changes support and mirror student reports.
	(Barr-Wilson & Roberts, 2016)	All participants reported the program positively influenced their body image during and immediately after their course. Most participants (9 out of 13) reported that the positive influence on their body image persisted up to 3 years post-course. Key positive influences were identified as: instructors (as role models), other girls on the course (creating a supportive, non-judgmental environment), and the natural environment. Participants defined “healthy living” holistically, including physical health, relationship with self, relationships with others, and emotional health.
	(Bateman & Waters, 2018)	Children developed confidence, autonomy, and resilience through scaffolded risk-taking. High levels of wellbeing and engagement were observed, reflected in enthusiasm, persistence, and joyful participation. Teacher–child interactions in outdoor environments promoted physical sustained shared thinking, fostering both resilience and wellbeing. Teachers’ subtle scaffolding, positioning, and shared play encouraged children’s independence, risk-taking, and emotional regulation.
	(Blaine & Akhurst, 2020)	Statistically significant increases in life effectiveness and resilience post-intervention, maintained at 4-month follow-up. No significant change in emotional literacy. Qualitative data highlighted benefits in friendships, self-awareness, resilience, leadership, and social skills. Some participants found the programme overly challenging; a strength-based approach was recommended.
	(Blum et al., 2025)	Participants reported significant mean increases across all Positive Youth Development assets (Caring, Connection, Contribution, Competence, Character, Confidence, and Happiness), including moderate effect sizes for all measures except Happiness. In addition, over 70% of the participants would recommend the program and/or do it again, suggesting program satisfaction.
	(Bradley & Male, 2017)	All three groups (children, parents, professionals) highlighted learning outcomes and the benefits of challenge and risk-taking. A key theme for children was the opportunity to make and talk about friends, which is significant given the social communication deficits associated with Autism Spectrum Disorder. Children were able to articulate and depict a range of subtle emotions in response to Forest School. Parents and professionals reported observing positive changes, including increased physical skills, tolerance for mess, confidence, and independence.
	(Brussoni et al., 2017)	Quality of Play Space: Seven Cs scores more than doubled in both centres, indicating significant quality improvement. Play/Social Behaviour: Significant decreases in depressed affect (mental health) and antisocial behaviour. Significant increases in play with natural materials, independent play (implied), and prosocial behaviour (Centre A only). Physical Activity: Significant decrease in moderate to vigorous physical activity. Risky play did not increase significantly. Spatial Use: Children increased their use of natural loose and fixed elements and used different and more areas of the play space.
	(Card & Burke, 2021)	Outdoor Kindergarten successfully meets and exceeds traditional kindergarten curriculum outcomes. It fosters advanced critical thinking, problem-solving, and collaborative skills through child-led, land-based inquiry. The model supports the development of a strong sense of self, place, and community. It is presented as a particularly valuable model for Indigenous communities to help reconnect children with their land and culture.
	(Davidson & Foster, 2024)	Outdoor Adventure Education significantly enhanced grit, mastery, and emotional regulation, though effects varied by demographic factors. Older, higher-SES, and white participants experienced greater growth, while relatedness showed minimal change. Authors concluded that purposefully designed Outdoor Adventure Education programmes using PERMA (positive emotions, engagement, relationships, meaning, and accomplishment) elements can effectively foster grit, resilience, and emotional competence.
	(Down et al., 2023)	The most valued aspects of Outdoor Adventure Education were opportunities to develop relationships, build social connections, self-efficacy, resilience, and a sense of individual empowerment. Students valued autonomy and freedom, which presented a challenge for teachers managing risk. Students desired staff who were relatable, approachable, and different from a typical classroom teacher. While adrenaline activities were popular, slower, journey-based activities were also highly valued for fostering connection and reflection.
	(Friedman et al., 2024)	Forest School provided benefits through play, autonomy, and development of practical, motor, and social skills. It was perceived as an exciting, freeing break from the typical school day. Success was contingent on adherence to routines (e.g., fire, food) and the supportive, knowledgeable attitudes of the adults present. Challenges included children absconding, peer conflict, and the intervention not being suitable for all children all the time. Forest School can support autonomy, competence, and relatedness
	(Haywood-Bird, 2017)	Children exercised personal power and agency through their outdoor play activities. Key examples of this powerful play included: Physically risky activities, such as climbing trees, complex imaginative play where they co-created roles and stories, the autonomous choice to play alone. The outdoor setting enabled a flexible “give and take” of power, letting children lead, follow, make choices, or withdraw from play seamlessly. Children independently assessed and acted within their own comfort levels for risk and physical ability. The open, natural environment fostered intricate social dramas that were initiated and developed by the children themselves, without adult guidance.
	(Little, 2022)	Increased physical competence, confidence, and problem-solving among toddlers. Educators developed greater trust in children’s abilities and became more comfortable with risk. The environment supported language, social, and motor skill development and encouraged educator reflection on pedagogical risk-taking. Toddlers engaged confidently in play involving heights, balance, speed, and seclusion, particularly using the tyre tower and rocks. Educators initially expressed safety concerns, but observations revealed that children managed risks effectively and were more capable than expected. Over time, educators’ attitudes shifted toward risk-positive pedagogies, reinforcing children’s agency and independence.
	(Okur-Berberoglu, 2021)	Unstructured outdoor play fostered five core developmental outcomes: observation, exploration, cognitive development, creativity, and self-confidence.
	(Orson et al., 2020)	Youth learned through active processes of struggling with challenges, including building perseverance, constructing positive mindsets, and solving social problems. Peers provided critical on-the-spot instrumental and emotional support that helped youth overcome challenges and facilitated learning. A positive group culture of compassion and mutual commitment, cultivated by instructors, was a key catalyst for the effective peer support.
	(Richmond et al., 2018)	Social Connectedness: The dominant outcome. Shared challenges and being away from technology fostered stronger bonds among students and between students and faculty. Self-Efficacy in Leadership: Students built confidence in leadership through taking on new roles, student-directed decision-making, reflection, and coaching from instructors. Recalibrated Sense of Self: Students reevaluated their capabilities and values, learning to be “comfortable being uncomfortable,” and gained a sense of empowerment and accomplishment. School Impact: Returning to a shared school environment allowed these benefits to be reinforced and sustained through continued relationships, shared narratives, and a supportive school culture.
	(Rocca, 2022)	Forest School promoted key areas of wellbeing, including social skills, emotional control, self-confidence, independence, creativity, and a bond with nature. Children built stronger friendships, resilience, and feelings of belonging and capability. The program’s benefits were categorized into eight wellbeing pathways: Social skills, emotional skills, risk management, autonomy and choice, cognitive development, play and creativity, bond with and care for nature, physical skills. The study found that Forest School boosts wellbeing by providing autonomy, a safe community, a connection to the environment, and chances to build skills and reflect.
	(Slee & Allan, 2019)	Significant difference in psychological well-being, statistically sig. differences in autonomy and relatedness. Collaborative effort and support for others continuously reinforced in qual. findings, supporting future challenge participants faced when transitioning schools. Teachers’ perspectives emphasised children’s freedom to plan and explore, undertake supported risk-taking and review naturally emerging experiences. Behaviours showed a degree of resonance 4 mths later in student and teacher discussions. Outdoor adventure residential programme exposure which helps pupils to (i) feel proud and content (well-being) (ii) become independent (autonomy), (iii) be good at something (competence) and (iv) feel valued as a group member (relatedness) can produce a range of adaptive capabilities that help transition to secondary school.
	(Tangen et al., 2022)	Toddlers can assess and managing risks through direct (e.g., slowing pace, careful looking) and indirect (observing peers) strategies. They handle risks sensibly and are drawn to challenging elements like slopes and water. Adult intervention, while sometimes necessary, can override a child’s own risk assessment and limit their learning opportunities. Free exploration in varied natural environments allows toddlers to develop risk competence.
	(Talley et al., 2023)	Five themes: Relationship & Community, Perseverance & Resiliency, Enjoyment & Finding Beauty in Nature, Leadership & Confidence, and Individual Growth. Participation in long-term outdoor adventure program built self-confidence and provided tools for a positive future.
	(Tsikalas & Martin, 2015)	Both the breadth (variety) and intensity (frequency) of outdoor exposure were positively associated with challenge seeking. For younger girls (Juniors, grades 4–5), the breadth of outdoor experiences was a stronger predictor of challenge seeking. For older girls (Cadettes, grades 6–8), the intensity (monthly participation) of outdoor experiences was a stronger predictor. Self-esteem was a strong predictor of challenge seeking for all girls. Girls who participated in outdoor activities monthly were significantly more likely to report high levels of challenge seeking and to agree that “Because of Girl Scouts, I learned to do things I thought I could not do.”
	(Tucker et al., 2016)	Mental health improvements across all Youth-Outcome Questionnaire domains (intrapersonal distress, social problems, behavioural dysfunction, etc.). Wilderness therapy participants moved toward healthier Body Mass Index levels and demonstrated clinically significant mental health improvements by discharge
	(Whittington & Aspelmeier, 2018)	Girls in all program types showed a small but significant increase in overall resilience. Adventure Education (AE) programs showed the greatest increases in resilience, positive peer relationships, and confidence. Girls in Experiential Education, Mixed, and AE programs reported significant improvements in positive peer relationships, while those in traditional camp settings did not. All program types led to significant increases in confidence, with the largest change again in AE programs.
	(I. R. Williams et al., 2018)	The study found no evidence of universal, positive effects on the 16 measured outcomes of wellbeing. Of the 16 scales, 14 showed no meaningful difference between the camp and control groups. The two scales that showed statistically significant differences (Basic Psychological Needs Scale-Relatedness and Strengths and Difficulties Questionnaire-Difficulties) had effects that were very small in magnitude. In contrast, qualitative data from student journals and reflections indicated that the program was “impactful and positive for some students,” with reports of increased independence, teamwork, self-awareness, and confidence.
Wellbeing (21)	(Allan et al., 2025)	Significant increases in resilience (36.33%) and well-being (23.12%) from pre- to post-test. These gains were largely retained at the one-month follow-up. The most powerful predictors of positive change were: being inspired by the countryside, solving one’s own problems, and having freedom of choice. Participants reported developing a broad array of 21st Century Skills.
	(Barfield et al., 2021)	Increasing Health-Related Competencies: Students increased their physical activity, improved their sleep, perceived less stress, and reported changes in dietary habits and electronic use. Increasing Social Relatedness: Students made new friends, felt more connected, and spent less time home alone after school. Increasing Autonomy and Intrinsic Motivation: Students recognized their emerging capabilities, and their increased confidence stimulated more action-oriented behaviour. Parent-perceived changes support and mirror student reports.
	(Bateman & Waters, 2018)	Children developed confidence, autonomy, and resilience through scaffolded risk-taking. High levels of wellbeing and engagement were observed, reflected in enthusiasm, persistence, and joyful participation. Teacher–child interactions in outdoor environments promoted physical sustained shared thinking, fostering both resilience and wellbeing. Teachers’ subtle scaffolding, positioning, and shared play encouraged children’s independence, risk-taking, and emotional regulation.
	(Blum et al., 2025)	Participants reported significant mean increases across all Positive Youth Development assets (Caring, Connection, Contribution, Competence, Character, Confidence, and Happiness), including moderate effect sizes for all measures except Happiness. In addition, over 70% of the participants would recommend the program and/or do it again, suggesting program satisfaction.
	(Bradley & Male, 2017)	All three groups (children, parents, professionals) highlighted learning outcomes and the benefits of challenge and risk-taking. A key theme for children was the opportunity to make and talk about friends, which is significant given the social communication deficits associated with Autism Spectrum Disorder. Children were able to articulate and depict a range of subtle emotions in response to Forest School. Parents and professionals reported observing positive changes, including increased physical skills, tolerance for mess, confidence, and independence.
	(Brussoni et al., 2017)	Quality of Play Space: Seven Cs scores more than doubled in both centres, indicating significant quality improvement. Play/Social Behaviour: Significant decreases in depressed affect (mental health) and antisocial behaviour. Significant increases in play with natural materials, independent play (implied), and prosocial behaviour (Centre A only). Physical Activity: Significant decrease in moderate to vigorous physical activity. Risky play did not increase significantly. Spatial Use: Children increased their use of natural loose and fixed elements and used different and more areas of the play space.
	(Down et al., 2023)	The most valued aspects of Outdoor Adventure Education were opportunities to develop relationships, build social connections, self-efficacy, resilience, and a sense of individual empowerment. Students valued autonomy and freedom, which presented a challenge for teachers managing risk. Students desired staff who were relatable, approachable, and different from a typical classroom teacher. While adrenaline activities were popular, slower, journey-based activities were also highly valued for fostering connection and reflection.
	(Friedman et al., 2024)	Forest School provided benefits through play, autonomy, and development of practical, motor, and social skills. It was perceived as an exciting, freeing break from the typical school day. Success was contingent on adherence to routines (e.g., fire, food) and the supportive, knowledgeable attitudes of the adults present. Challenges included children absconding, peer conflict, and the intervention not being suitable for all children all the time. Forest School can support autonomy, competence, and relatedness.
	(Hinchion et al., 2021)	Children described risky play as “scary,” “exciting,” and fun. Eight categories of risky play were identified, including established categories (heights, speed) and new ones for this age group (risky construction, breaking rules). Risky play was a highly social and subjective experience, influenced by peers and the environment. Children demonstrated autonomy by navigating and sometimes circumventing adult rules to engage in meaningful risky play.
	(Lazaridis et al., 2023)	The programme successfully promoted adolescents’ basic psychological needs. Students reported increased feelings of autonomy (freedom of choice), competence (self-confidence and capability), and relatedness (improved peer relationships and teamwork). Notably, the programme provided girls with a greater sense of autonomy and competence compared to typical physical education lessons.
	(Mackenzie et al., 2018)	Participants’ physical activity (steps per day) increased by 121% during the outdoor adventure-based science curriculum compared to school. All psychological measures (flow, intrinsic motivation, autonomy, competence, relatedness, enjoyment, learning climate) were significantly higher during the outdoor adventure science curriculum (OASC) than in pre- and post-school settings. No significant changes were found in physical activity attitudes or active outdoor identity, likely due to high baseline scores and the short program duration. The OASC successfully created an autonomy-supportive learning environment that enhanced engagement and motivation for both science and physical activity.
	(Richmond & Sibthorp, 2019)	Increased self-efficacy for dealing with challenge; significant association between increased self-efficacy for using help-seeking behaviour; significant association between Outdoor Adventure Education and sense of belonging at school; no significant change in student growth mindsets toward leadership or emotional regulation; physically and emotionally challenging backcountry setting was the key mechanism that fostered growth, compelling students to rely on one another and instructors.
	(Richmond et al., 2018)	Social Connectedness: The dominant outcome. Shared challenges and being away from technology fostered stronger bonds among students and between students and faculty. Self-Efficacy in Leadership: Students built confidence in leadership through taking on new roles, student-directed decision-making, reflection, and coaching from instructors. Recalibrated Sense of Self: Students reevaluated their capabilities and values, learning to be “comfortable being uncomfortable,” and gained a sense of empowerment and accomplishment. School Impact: Returning to a shared school environment allowed these benefits to be reinforced and sustained through continued relationships, shared narratives, and a supportive school culture.
	(Rocca, 2022)	Forest School promoted key areas of wellbeing, including social skills, emotional control, self-confidence, independence, creativity, and a bond with nature. Children built stronger friendships, resilience, and feelings of belonging and capability. The program’s benefits were categorized into eight wellbeing pathways: Social skills, emotional skills, risk management, autonomy and choice, cognitive development, play and creativity, bond with and care for nature, physical skills. The study found that Forest School boosts wellbeing by providing autonomy, a safe community, a connection to the environment, and chances to build skills and reflect.
	(Scrutton, 2015)	Pupils who perceived themselves as having poorer personal and social skills at the outset gained the most benefit and retained it, while higher-scoring pupils lost their gains.
	(Scogin et al., 2025)	Positive, statistically significant growth in almost all social-emotional skill areas tested; Academic Outcomes: 57.1% of children were at or above the early literacy benchmark when tested in kindergarten (caregivers reported an increase in academic readiness; no statistically significant changes in connections to nature (but mean scores shifted from a “neutral” to a “good” connection, and caregivers reported children had more interest in the outdoors)
	(Slee & Allan, 2019)	Significant difference in psychological well-being, statistically sig. differences in autonomy and relatedness. Collaborative effort and support for others continuously reinforced in qual. findings, supporting future challenge participants faced when transitioning schools. Teachers’ perspectives emphasised children’s freedom to plan and explore, undertake supported risk-taking and review naturally emerging experiences. Behaviours showed a degree of resonance 4 mths later in student and teacher discussions. Outdoor adventure residential programme exposure which helps pupils to (i) feel proud and content (well-being) (ii) become independent (autonomy), (iii) be good at something (competence) and (iv) feel valued as a group member (relatedness) can produce a range of adaptive capabilities that help transition to secondary school.
	(Talley et al., 2023)	Five themes: Relationship & Community, Perseverance & Resiliency, Enjoyment & Finding Beauty in Nature, Leadership & Confidence, and Individual Growth. Participation in long-term outdoor adventure program built self-confidence and provided tools for a positive future.
	(Tucker et al., 2016)	Mental health improvements across all Y-OQ domains (intrapersonal distress, social problems, behavioural dysfunction, etc.) Wilderness therapy participants moved toward healthier Body Mass Index levels and demonstrated clinically significant mental health improvements by discharge.
	(Whittington & Aspelmeier, 2018)	Girls in all program types showed a small but significant increase in overall resilience. Adventure Education (AE) programs showed the greatest increases in resilience, positive peer relationships, and confidence. Girls in Experiential Education, Mixed, and AE programs reported significant improvements in positive peer relationships, while those in traditional camp settings did not. All program types led to significant increases in confidence, with the largest change again in AE programs.
	(Zygmont & Naidoo, 2018)	Adolescents experienced a school-based adventure programme in four distinct ways ((a) long gruelling school hike, (b) school initiation/rite of passage programme, (c) once-in-a-lifetime group adventure, and (d) multifaceted learning and development opportunity), based on the variation in meanings of six aspects of the programme (a) programme characterisation, (b) the nature of group processes and interactions, (c) the nature and level of connection and interactions with adult group leaders, (d) the depth of engagement in various components of the programme.
Physical changes and skills (14)	(Barfield et al., 2021)	Increasing Health-Related Competencies: Students increased their physical activity, improved their sleep, perceived less stress, and reported changes in dietary habits and electronic use. Increasing Social Relatedness: Students made new friends, felt more connected, and spent less time home alone after school. Increasing Autonomy and Intrinsic Motivation: Students recognized their emerging capabilities, and their increased confidence stimulated more action-oriented behaviour. Parent-perceived changes support and mirror student reports.
	(Barr-Wilson & Roberts, 2016)	All participants reported the program positively influenced their body image during and immediately after their course. Most participants (9 out of 13) reported that the positive influence on their body image persisted up to 3 years post-course. Key positive influences were identified as: instructors (as role models), other girls on the course (creating a supportive, non-judgmental environment), and the natural environment. Participants defined “healthy living” holistically, including physical health, relationship with self, relationships with others, and emotional health.
	(Bateman & Waters, 2018)	Children developed confidence, autonomy, and resilience through scaffolded risk-taking. High levels of wellbeing and engagement were observed, reflected in enthusiasm, persistence, and joyful participation. Teacher–child interactions in outdoor environments promoted physical sustained shared thinking, fostering both resilience and wellbeing. Teachers’ subtle scaffolding, positioning, and shared play encouraged children’s independence, risk-taking, and emotional regulation.
	(Bradley & Male, 2017)	All three groups (children, parents, professionals) highlighted learning outcomes and the benefits of challenge and risk-taking. A key theme for children was the opportunity to make and talk about friends, which is significant given the social communication deficits associated with Autism Spectrum Disorder. Children were able to articulate and depict a range of subtle emotions in response to Forest School. Parents and professionals reported observing positive changes, including increased physical skills, tolerance for mess, confidence, and independence.
	(Brusaferro, 2020)	Children developed an ecological identity during nature play by making nature connections, mastering their bodies, feeling part of the forest preschool community, and using movement and senses
	(Brussoni et al., 2017)	Quality of Play Space: Seven Cs scores more than doubled in both centres, indicating significant quality improvement. Play/Social Behaviour: Significant decreases in depressed affect (mental health) and antisocial behaviour. Significant increases in play with natural materials, independent play (implied), and prosocial behaviour (Centre A only). Physical Activity: Significant decrease in moderate to vigorous physical activity. Risky play did not increase significantly. Spatial Use: Children increased their use of natural loose and fixed elements and used different and more areas of the play space.
	(Haywood-Bird, 2017)	Children exercised personal power and agency through their outdoor play activities. Key examples of this powerful play included: Physically risky activities, such as climbing trees, complex imaginative play where they co-created roles and stories, the autonomous choice to play alone. The outdoor setting enabled a flexible “give and take” of power, letting children lead, follow, make choices, or withdraw from play seamlessly. Children independently assessed and acted within their own comfort levels for risk and physical ability. The open, natural environment fostered intricate social dramas that were initiated and developed by the children themselves, without adult guidance.
	(Hinchion et al., 2021)	Children described risky play as “scary,” “exciting,” and fun. Eight categories of risky play were identified, including established categories (heights, speed) and new ones for this age group (risky construction, breaking rules). Risky play was a highly social and subjective experience, influenced by peers and the environment. Children demonstrated autonomy by navigating and sometimes circumventing adult rules to engage in meaningful risky play.
	(Little, 2022)	Increased physical competence, confidence, and problem-solving among toddlers. Educators developed greater trust in children’s abilities and became more comfortable with risk. The environment supported language, social, and motor skill development and encouraged educator reflection on pedagogical risk-taking. Toddlers engaged confidently in play involving heights, balance, speed, and seclusion, particularly using the tyre tower and rocks. Educators initially expressed safety concerns, but observations revealed that children managed risks effectively and were more capable than expected. Over time, educators’ attitudes shifted toward risk-positive pedagogies, reinforcing children’s agency and independence.
	(Mackenzie et al., 2018)	Participants’ physical activity (steps per day) increased by 121% during the outdoor adventure-based science curriculum compared to school. All psychological measures (flow, intrinsic motivation, autonomy, competence, relatedness, enjoyment, learning climate) were significantly higher during the outdoor adventure science curriculum (OASC) than in pre- and post-school settings. No significant changes were found in physical activity attitudes or active outdoor identity, likely due to high baseline scores and the short program duration. The OASC successfully created an autonomy-supportive learning environment that enhanced engagement and motivation for both science and physical activity.
	(Richmond & Sibthorp, 2019)	Increased self-efficacy for dealing with challenge; significant association between increased self-efficacy for using help-seeking behaviour; significant association between Outdoor Adventure Education and sense of belonging at school; no significant change in student growth mindsets toward leadership or emotional regulation; physically and emotionally challenging backcountry setting was the key mechanism that fostered growth, compelling students to rely on one another and instructors.
	(Rocca, 2022)	Forest School promoted key areas of wellbeing, including social skills, emotional control, self-confidence, independence, creativity, and a bond with nature. Children built stronger friendships, resilience, and feelings of belonging and capability. The program’s benefits were categorized into eight wellbeing pathways: Social skills, emotional skills, risk management, autonomy and choice, cognitive development, play and creativity, bond with and care for nature, physical skills. The study found that Forest School boosts wellbeing by providing autonomy, a safe community, a connection to the environment, and chances to build skills and reflect.
	(Stoica et al., 2025)	The program led to statistically significant improvements in motor skills. Significant improvements were observed in dynamic balance and general coordination for most groups. Physical fitness scores improved significantly, showing a decrease in the mean score (indicating better fitness) for all groups. Improvements varied by age and gender, with boys and different age groups showing distinct patterns of progress in different skills.
	(Tucker et al., 2016)	Mental health improvements across all Youth-Outcome Questionnaire domains (intrapersonal distress, social problems, behavioural dysfunction, etc.). Wilderness therapy participants moved toward healthier Body Mass Index levels and demonstrated clinically significant mental health improvements by discharge
Autonomy (10)	(Bateman & Waters, 2018)	Children developed confidence, autonomy, and resilience through scaffolded risk-taking. High levels of wellbeing and engagement were observed, reflected in enthusiasm, persistence, and joyful participation. Teacher–child interactions in outdoor environments promoted physical sustained shared thinking, fostering both resilience and wellbeing. Teachers’ subtle scaffolding, positioning, and shared play encouraged children’s independence, risk-taking, and emotional regulation.
	(Barfield et al., 2021)	Increasing Health-Related Competencies: Students increased their physical activity, improved their sleep, perceived less stress, and reported changes in dietary habits and electronic use. Increasing Social Relatedness: Students made new friends, felt more connected, and spent less time home alone after school. Increasing Autonomy and Intrinsic Motivation: Students recognized their emerging capabilities, and their increased confidence stimulated more action-oriented behaviour. Parent-perceived changes support and mirror student reports.
	(Down et al., 2023)	The most valued aspects of Outdoor Adventure Education were opportunities to develop relationships, build social connections, self-efficacy, resilience, and a sense of individual empowerment. Students valued autonomy and freedom, which presented a challenge for teachers managing risk. Students desired staff who were relatable, approachable, and different from a typical classroom teacher. While adrenaline activities were popular, slower, journey-based activities were also highly valued for fostering connection and reflection.
	(Friedman et al., 2024)	Forest School provided benefits through play, autonomy, and development of practical, motor, and social skills. It was perceived as an exciting, freeing break from the typical school day. Success was contingent on adherence to routines (e.g., fire, food) and the supportive, knowledgeable attitudes of the adults present. Challenges included children absconding, peer conflict, and the intervention not being suitable for all children all the time. Forest School can support autonomy, competence, and relatedness.
	(Haywood-Bird, 2017)	Children exercised personal power and agency through their outdoor play activities. Key examples of this powerful play included: Physically risky activities, such as climbing trees, complex imaginative play where they co-created roles and stories, the autonomous choice to play alone. The outdoor setting enabled a flexible “give and take” of power, letting children lead, follow, make choices, or withdraw from play seamlessly. Children independently assessed and acted within their own comfort levels for risk and physical ability. The open, natural environment fostered intricate social dramas that were initiated and developed by the children themselves, without adult guidance.
	(Hinchion et al., 2021)	Children described risky play as “scary,” “exciting,” and fun. Eight categories of risky play were identified, including established categories (heights, speed) and new ones for this age group (risky construction, breaking rules). Risky play was a highly social and subjective experience, influenced by peers and the environment. Children demonstrated autonomy by navigating and sometimes circumventing adult rules to engage in meaningful risky play.
	(Lazaridis et al., 2023)	The programme successfully promoted adolescents’ basic psychological needs. Students reported increased feelings of autonomy (freedom of choice), competence (self-confidence and capability), and relatedness (improved peer relationships and teamwork). Notably, the programme provided girls with a greater sense of autonomy and competence compared to typical physical education lessons.
	(Rocca, 2022)	Forest School promoted key areas of wellbeing, including social skills, emotional control, self-confidence, independence, creativity, and a bond with nature. Children built stronger friendships, resilience, and feelings of belonging and capability. The program’s benefits were categorized into eight wellbeing pathways: Social skills, emotional skills, risk management, autonomy and choice, cognitive development, play and creativity, bond with and care for nature, physical skills. The study found that Forest School boosts wellbeing by providing autonomy, a safe community, a connection to the environment, and chances to build skills and reflect.
	(Slee & Allan, 2019)	Significant difference in psychological well-being, statistically sig. differences in autonomy and relatedness. Collaborative effort and support for others continuously reinforced in qual. findings, supporting future challenge participants faced when transitioning schools. Teachers’ perspectives emphasised children’s freedom to plan and explore, undertake supported risk-taking and review naturally emerging experiences. Behaviours showed a degree of resonance 4 mths later in student and teacher discussions. Outdoor adventure residential programme exposure which helps pupils to (i) feel proud and content (well-being) (ii) become independent (autonomy), (iii) be good at something (competence) and (iv) feel valued as a group member (relatedness) can produce a range of adaptive capabilities that help transition to secondary school.
	(Tangen et al., 2022)	Toddlers can assess and managing risks through direct (e.g., slowing pace, careful looking) and indirect (observing peers) strategies. They handle risks sensibly and are drawn to challenging elements like slopes and water. Adult intervention, while sometimes necessary, can override a child’s own risk assessment and limit their learning opportunities. Free exploration in varied natural environments allows toddlers to develop risk competence.
Nature connectedness (9)	(Brusaferro, 2020)	Children developed an ecological identity during nature play by making nature connections, mastering their bodies, feeling part of the forest preschool community, and using movement and senses
	(Davidson & Foster, 2024)	Outdoor Adventure Education significantly enhanced grit, mastery, and emotional regulation, though effects varied by demographic factors. Older, higher-SES, and white participants experienced greater growth, while relatedness showed minimal change. Authors concluded that purposefully designed Outdoor Adventure Education programmes using PERMA (positive emotions, engagement, relationships, meaning, and accomplishment) elements can effectively foster grit, resilience, and emotional competence.
	(Deniz & Cevher Kalburan, 2024)	High-speed play most frequent play type, followed by play at great heights; German preschool had more risky play opportunities than Turkey; educator attitude, mixed-age groups, allocated free playtime influenced risky play experiences
	(Down et al., 2023)	The most valued aspects of Outdoor Adventure Education were opportunities to develop relationships, build social connections, self-efficacy, resilience, and a sense of individual empowerment. Students valued autonomy and freedom, which presented a challenge for teachers managing risk. Students desired staff who were relatable, approachable, and different from a typical classroom teacher. While adrenaline activities were popular, slower, journey-based activities were also highly valued for fostering connection and reflection.
	(Olsen et al., 2025)	Toddlers engage in a wide range of activities, including gross motor, loose material, and water activities in the natural environment. There were large variations among the toddlers concerning time spent on different surfaces and exploratory activities, supporting the notion of nature as a child-friendly environment for toddlers
	(Rocca, 2022)	Forest School promoted key areas of wellbeing, including social skills, emotional control, self-confidence, independence, creativity, and a bond with nature. Children built stronger friendships, resilience, and feelings of belonging and capability. The program’s benefits were categorized into eight wellbeing pathways: Social skills, emotional skills, risk management, autonomy and choice, cognitive development, play and creativity, bond with and care for nature, physical skills. The study found that Forest School boosts wellbeing by providing autonomy, a safe community, a connection to the environment, and chances to build skills and reflect.
	(Scogin et al., 2025)	Positive, statistically significant growth in almost all social-emotional skill areas tested; Academic Outcomes: 57.1% of children were at or above the early literacy benchmark when tested in kindergarten (caregivers reported an increase in academic readiness; no statistically significant changes in connections to nature (but mean scores shifted from a “neutral” to a “good” connection, and caregivers reported children had more interest in the outdoors)
	(Slee & Allan, 2019)	Significant difference in psychological well-being, statistically sig. differences in autonomy and relatedness. Collaborative effort and support for others continuously reinforced in qual. findings, supporting future challenge participants faced when transitioning schools. Teachers’ perspectives emphasised children’s freedom to plan and explore, undertake supported risk-taking and review naturally emerging experiences. Behaviours showed a degree of resonance 4 mths later in student and teacher discussions. Outdoor adventure residential programme exposure which helps pupils to (i) feel proud and content (well-being) (ii) become independent (autonomy), (iii) be good at something (competence) and (iv) feel valued as a group member (relatedness) can produce a range of adaptive capabilities that help transition to secondary school.
	(Talley et al., 2023)	Five themes: Relationship & Community, Perseverance & Resiliency, Enjoyment & Finding Beauty in Nature, Leadership & Confidence, and Individual Growth. Participation in long-term outdoor adventure program built self-confidence and provided tools for a positive future.
Quality of play and adventure education provision (4)	(Brussoni et al., 2017)	Quality of Play Space: Seven Cs scores more than doubled in both centres, indicating significant quality improvement. Play/Social Behaviour: Significant decreases in depressed affect (mental health) and antisocial behaviour. Significant increases in play with natural materials, independent play (implied), and prosocial behaviour (Centre A only). Physical Activity: Significant decrease in moderate to vigorous physical activity. Risky play did not increase significantly. Spatial Use: Children increased their use of natural loose and fixed elements and used different and more areas of the play space.
	(Haywood-Bird, 2017)	Children exercised personal power and agency through their outdoor play activities. Key examples of this powerful play included: Physically risky activities, such as climbing trees, complex imaginative play where they co-created roles and stories, the autonomous choice to play alone. The outdoor setting enabled a flexible “give and take” of power, letting children lead, follow, make choices, or withdraw from play seamlessly. Children independently assessed and acted within their own comfort levels for risk and physical ability. The open, natural environment fostered intricate social dramas that were initiated and developed by the children themselves, without adult guidance.
	(Hinchion et al., 2021)	Children described risky play as “scary,” “exciting,” and fun. Eight categories of risky play were identified, including established categories (heights, speed) and new ones for this age group (risky construction, breaking rules). Risky play was a highly social and subjective experience, influenced by peers and the environment. Children demonstrated autonomy by navigating and sometimes circumventing adult rules to engage in meaningful risky play.
	(Little, 2022)	Increased physical competence, confidence, and problem-solving among toddlers. Educators developed greater trust in children’s abilities and became more comfortable with risk. The environment supported language, social, and motor skill development and encouraged educator reflection on pedagogical risk-taking. Toddlers engaged confidently in play involving heights, balance, speed, and seclusion, particularly using the tyre tower and rocks. Educators initially expressed safety concerns, but observations revealed that children managed risks effectively and were more capable than expected. Over time, educators’ attitudes shifted toward risk-positive pedagogies, reinforcing children’s agency and independence.
Participants influence educators (3)	(Little, 2022)	Increased physical competence, confidence, and problem-solving among toddlers. Educators developed greater trust in children’s abilities and became more comfortable with risk. The environment supported language, social, and motor skill development and encouraged educator reflection on pedagogical risk-taking. Toddlers engaged confidently in play involving heights, balance, speed, and seclusion, particularly using the tyre tower and rocks. Educators initially expressed safety concerns, but observations revealed that children managed risks effectively and were more capable than expected. Over time, educators’ attitudes shifted toward risk-positive pedagogies, reinforcing children’s agency and independence.
	(Slee & Allan, 2019)	Significant difference in psychological well-being, statistically sig. differences in autonomy and relatedness. Collaborative effort and support for others continuously reinforced in qual. findings, supporting future challenge participants faced when transitioning schools. Teachers’ perspectives emphasised children’s freedom to plan and explore, undertake supported risk-taking and review naturally emerging experiences. Behaviours showed a degree of resonance 4 mths later in student and teacher discussions. Outdoor adventure residential programme exposure which helps pupils to (i) feel proud and content (well-being) (ii) become independent (autonomy), (iii) be good at something (competence) and (iv) feel valued as a group member (relatedness) can produce a range of adaptive capabilities that help transition to secondary school.
	(Tangen et al., 2022)	Toddlers can assess and managing risks through direct (e.g., slowing pace, careful looking) and indirect (observing peers) strategies. They handle risks sensibly and are drawn to challenging elements like slopes and water. Adult intervention, while sometimes necessary, can override a child’s own risk assessment and limit their learning opportunities. Free exploration in varied natural environments allows toddlers to develop risk competence.

## Data Availability

The data necessary to reproduce the analyses presented here are publicly accessible—data are available from the first author upon reasonable request. The analytic code necessary to reproduce the analyses presented in this paper is publicly accessible—code is available from the first author. The materials necessary to attempt to replicate the findings presented here are publicly accessible—materials are available from the first author. The analyses presented here were not preregistered.

## Supplementary Materials

The following supporting information can be downloaded at: https://www.mdpi.com/article/10.3390/bs16010005/s1, File S1: PRISMA-ScR Checklist. File S2: Search Terms and Subject or Field Codes for Each Database.
