# Supplementary material for: Risky Outdoor Play and Adventure Education in Nature for Child and Adolescent Wellbeing: A Scoping Review
Source: Behav Sci (Basel). 2025 Dec 19;16(1):5. doi: 10.3390/bs16010005 (PMC12837311; doi:10.3390/bs16010005)
Supplement: Supplementary file 1 [file behavsci-16-00005-s001.zip › Supplementary File S2. Search Terms and Subject or Field Codes for Each Database.pdf]

Complete Search Strategy for ProQuest Central – conducted 16/7/2025
Language = English, and Publication Date = 2015 onwards

Search #	Search Terms	Results
S1	(child* OR adolescen*) OR Mainsubject (Children AND youth)	3,980,492
S2	(Risk* OR Challeng* OR Adventur*) OR Subject (risk)	6,794,240
S3	(outdoor OR natur*) OR subject (outdoor education OR nature OR play)	2,913,271
S4	(educat* OR school*) OR subject (Early childhood education OR Elementary education OR secondary education)	7,407,956
S5	(benefit* OR improve* OR development* OR restor* OR Brain OR Cognitive OR Neuro* OR Social OR biophysic* OR Self-regulation OR Holistic OR wholistic OR Resilien* OR Confiden* OR Calm* OR brave* OR esteem OR Identity OR engage* OR wellbeing OR well-being OR "Mental health") OR subject(self concept OR self esteem OR physical activity OR academic achievement))	17,209,616
S6	S1 AND S2 AND S3 AND S4 AND S5	2826

Complete Search Strategy for Academic Search Complete (EBSCO) – conducted 16/7/2025
Language = English, and Publication Date = 2015 onwards

Search #	Search Terms	Results
S1	XB child* OR adolescen* OR youth OR SU children OR adolescence	874,595
S2	XB (risk* OR challeng* OR adventur*) OR SU (risk)	2,480,747
S3	XB (outdoor OR natur*) OR SU (Outdoor education OR adventure education OR nature OR imaginative play)	1,047,413
S4	XB school* OR XB educat* OR SU (Early childhood education OR Primary education OR Elementary education OR secondary education)	762,120
S5	XB (benefit* OR physical OR cognitive OR psychosocial OR psycho-social OR develop* OR improve* OR restor* OR brain OR neuro* OR social OR biophysic* OR self?regulation OR holistic OR wholistic OR resilien* OR confidence OR calm* OR brave* OR identity OR engage* OR well?being OR "mental health") OR SU (Self-perception OR Academic achievement) OR SU (physical activity OR well-being OR Psychological resilience)	7,153,260
S6	S1 AND S2 AND S3 AND S4 AND S5	2065

Complete Search Strategy for Education Research Complete (EBSCO) – conducted 16/7/2025
Language = English, and Publication Date = 2015 onwards

Search #	Search Terms	Results
S1	TI (child* OR adolescen* OR youth) OR AB (child* OR adolescen* OR youth) OR SU c(hildren OR adolescence)	721,375
S2	TI (risk* OR challeng* OR adventur*) OR AB (risk* OR challeng* OR adventur*)	185,074
S3	TI (outdoor OR natur*) OR AB (outdoor OR natur*) OR SU (outdoor education OR adventure education)	58,190
S4	TI (educat* OR school*) OR AB (educat* OR school*) OR SU (Early childhood education OR Primary education OR Elementary education OR secondary education)	454,455
S5	TI (benefit* OR physical OR cognitive OR psychosocial OR psycho-social OR develop* OR improve* OR restor* OR brain OR neuro* OR social OR biophysic* OR self?regulation OR holistic OR wholistic OR resilien* OR confidence OR calm* OR brave* OR identity OR engage* OR well?being OR "mental health") OR AB (benefit* OR physical OR cognitive OR psychosocial OR psycho-social OR develop* OR improve* OR restor* OR brain OR neuro* OR social OR biophysic* OR self?regulation OR holistic OR wholistic OR resilien* OR confidence OR calm* OR brave* OR identity OR engage* OR well?being OR "mental health") OR SU autonomy (psychology) OR SU (self-esteem OR Academic improvement OR exercise OR health promotion OR psychological resilience)	601,467
S6	S1 AND S2 AND S3 AND S4 AND S5	1008

Complete Search Strategy for APA PsychInfo (EBSCO) – conducted 16/7/2025
Language = English, and Publication Date = 2015 onwards

Search #	Search Terms	Results
S1	XB child* OR XB adolescen* OR XB youth OR MJ childhood development OR MJ early adolescence	339,610
S2	XB (risk* OR challeng* OR adventur*) OR MJ Experiential learning	411,680
S3	XB (outdoor OR natur*)	118,071
S4	XB educat* OR XB school* OR SU (Early childhood education OR Primary school students OR secondary education OR Elementary school students OR Elementary education OR secondary education)	336,752
S5	XB benefit* OR physical OR cognitive OR psychosocial OR psycho-social OR develop* OR improve* OR restor* OR brain OR neuro* OR social OR biophysic* OR self?regulation OR holistic OR wholistic OR resilien* OR confidence OR calm* OR brave* OR identity OR engage* OR well?being OR "mental health" OR MJ (Self-perception OR	1,573,676

	Academic achievement OR exercise OR well being OR MJ Resilience (psychological) OR Pretend play OR risk taking	
S6	S1 AND S2 AND S3 AND S4 AND S5	1830

Complete Search Strategy for ERIC (ProQuest) – conducted 16/7/2025
Language = English, and Publication Date = 2015 onwards

Search #	Search Terms	Results
S1	mainsubject(Children AND youth) OR abstract(child* OR adolescen*) OR title(child* OR adolescen*)	69,223
S2	abstract(Risk* OR Challeng* OR Adventur*) OR title(Risk* OR Challeng* OR Adventur*) OR subject(risk)	75,207
S3	abstract(outdoor OR natur*) OR title(outdoor OR natur*) OR subject(outdoor education OR nature OR play)	28,937
S4	abstract(educat* OR school*) OR title(educat* OR school*) OR subject(Early childhood education OR Elementary education OR secondary education)	258,983
S5	abstract(benefit* OR improve* OR development* OR restor* OR Brain OR Cognitive OR Neuro* OR Social OR biophysic* OR Self-regulation OR Holistic OR wholistic OR Resilien* OR Confiden* OR Calm* OR brave* OR esteem OR Identity OR engage* OR wellbeing OR well-being OR "Mental health") OR title(benefit* OR improve* OR development* OR restor* OR Brain OR Cognitive OR Neuro* OR Social OR biophysic* OR Self-regulation OR Holistic OR wholistic OR Resilien* OR Confiden* OR Calm* OR brave* OR esteem OR Identity OR engage* OR well-being OR wellbeing OR "Mental health") OR subject(self concept OR self esteem OR physical activity OR academic achievement)	257,598
S6	S1 AND S2 AND S3 AND S4 AND S5	995
